# The Essential Oil-Bearing Plants in the United Arab Emirates (UAE): An Overview

**DOI:** 10.3390/molecules26216486

**Published:** 2021-10-27

**Authors:** Suzan Marwan Shahin, Abdul Jaleel, Mohammed Abdul Muhsen Alyafei

**Affiliations:** 1 Department of Integrative Agriculture, College of Agriculture and Veterinary Medicine, United Arab Emirates University, Al Ain 15551, United Arab Emirates; drsuzan.s@uaqu.ac.ae (S.M.S.); abdul.jaleel@uaeu.ac.ae (A.J.); 2 Research and Development Head, Umm Al Quwain University, Umm Al Quwain 536, United Arab Emirates

**Keywords:** arid lands, essential oil-bearing plants, indigenous and naturalized plants, United Arab Emirates (UAE)

## Abstract

Essential Oils (EOs) are expensive hydrocarbons produced exclusively by specific species in the plant kingdom. Their applications have deep roots in traditional herbal medicine, which lacks scientific evidence. Nowadays, more than ever, there is a growing global interest in research-based discoveries that maintain and promote health conditions. Consequently, EOs became a much attractive topic for both research and industry, with revenues reaching billions of dollars annually. In this work, we provide key guidance to all essential oil-bearing plants growing in the United Arab Emirates (UAE). The comprehensive data were collected following an extensive, up-to-date literature review. The results identified 137 plant species, including indigenous and naturalized ones, in the UAE, citing over 180 published research articles. The general overview included plant botanical names, synonyms, common names (Arabic and English), families and taxonomic authority. The study acts as a baseline and accelerator for research, industry and discoveries in multiple disciplines relying on essential oil-bearing plants.

## 1. Introduction

Globally, the essential oils (EOs) industry creates billions of dollars of revenue annually. Therefore, more attention has been given recently to this sector, as a natural primary resource for phytochemicals. Indeed, the EOs industry has a wide range of enormous applications in various fields, such as pharmaceuticals, aromatherapy, healthcare, cosmetics, food flavoring, food preservation and the fragrance industry [1].

Briefly, EOs are concentrated phytochemicals comprised mostly of terpenes, oxygenated terpenes, sesquiterpenes and oxygenated sesquiterpenes [2,3]. EOs are volatiles associated with a characteristic aroma resulting from the complex interaction between hundreds of volatiles. These hydrophobic compounds are produced exclusively from certain plant species as secondary metabolites, acting as defense phytochemicals [4].

In fact, EOs applications have deep roots in old traditional practices, in which they were a natural resource to treat infections and sicknesses for hundreds of years [3,4]. However, such traditional practices lack scientific validation, and thus have to be remarkably studied and tested, looking for scientific justification. The studies about essential oils from UAE plants and their biological activities are scanty when compared to other research in plant-based fields. There are studies such as extraction of essential oil of *Haplophyllum tuberculatum* [5], *Teucrium stocksianum* [6], *Pulicaria glutinosa* [7], *Cleome amblyocarpa* [4] and *Moringa peregrina* [8] from the United Arab Emirates.

The main objective of this work is to provide key guidance to all EO-bearing plants indigenous and naturalized to the United Arab Emirates (UAE), which included highlights on all available EO-bearing plant species, their families, botanical names, common names (Arabic and English) and taxonomic authority. It worth mentioning that this is the first record in the literature that provides the essential oil-bearing plants of the UAE. The value of such data will accelerate research, knowledge and discoveries in multiple disciplines (e.g., environment, biology, chemistry, chemical engineering, pharmacognosy, pharmacology and healthcare). The findings are key knowledge to justify the rich ethnomedicinal applications of the aromatic medicinal plants of the UAE. Additionally, this study will be supportive to decision-makers in strategic and sustainable planning for essential oil-bearing plants of the region.

## 2. Study Location

This work covered the UAE as a study location (land area of around 82,880 km^2^), which is located in the arid Western parts of Asia at the southeast end of the Arabian Peninsula on the Arabian/Persian Gulf (Latitude 22°30′ and 26°10′ N and longitude 51° and 56°25′ E). 

### Weather Conditions and Soil Analysis

The study location belongs to the arid zone; the climate is characterized by high summer temperatures (around 46 °C) and high humidity rates along the two coastal lines. It is characterized by a low and irregular precipitation rate (60 to 160 mm) [9].

The soil is classified as sandy sodic soil with a high permeability rate, low water holding capacity, low moisture content and low fertility rate [9,10]. Soil salinity is one of its major problems, especially in the coastal lines [11].

Conventional surface water resources include seasonal floods, springs and Falajes. The only groundwater resource comes from aquifers [12]. The high dependency on groundwater aquifers with low recharging rates causes both groundwater depletion and saline water intrusion, creating concerns that aquifer supplies may soon be depleted [13] and indicating a challenging future for the sustainability of the agricultural sector [12].

## 3. Data Collection Methodologies

To best of our knowledge, all existed references “online and hardcopy printed sources” related to the UAE flora were reviewed to collect the botanical names of all the UAE indigenous and naturalized plants, which were around 800 plant species. The references included Batanouny [14], Western [15], Tanira et al., [16], Wasfi [17], Karim [18], Emirates Natural History Group [19], Böer and Chaudhary [20], Jongbloed et al., [21], Brown and Sakkir [22], Aspinall [23], Zayed Complex for Herbal Research and Traditional Medicine (ZCHRTM) [24], Handa et al., [25], Karim and Dakheel [26], Mousa and Fawzi [27], Sakkir et al., [28], Fawzi and Ksiksi [29], Hurriez [30], Feulner [31], El-Keblawy et al., [32] and the Environmental Agency of Abu Dhabi [33,34].

After collecting the botanical names and synonyms of all documented Emirati indigenous and naturalized plants, each plant was subjected individually to an extensive literature review. The literature was collected using the online resource “Google Scholar”, in which all the published works indexed in “Scopus”, “Web of Science” and “PubMed”. Each plant was searched individually using the keywords “botanical name/synonyms + essential oil”. To the best of our knowledge, all existed published articles were carefully screened and over 180 of the latest articles were cited.

## 4. Results and Discussions

### 4.1. A Comprehensive Overview

All the indigenous and naturalized plants of the study location were evaluated and the result was establishing a full list of all Emirati EO-bearing plants (Table 1), including an overview of 137 Emirati EO-bearing plants belonging to 46 families, all cited based on up-to-date literature (over 180 references). Meaning that, EO-bearing plants comprise 17% of the estimated 800 indigenous and naturalized plants.

According to Raut and Karuppayil [185] there are around 2000 identified EO-bearing plants globally. Therefore, it is of high value that a country (with estimated 83 km^2^ land area and with limited freshwater resources) includes 137 EO-bearing plants. It is worth mentioning that although some of the UAE indigenous and naturalized plants have rich traditional therapeutic applications and belong to important medicinal families, there are no data yet available related to the potential of their EOs. Therefore, it is expected that many of the medicinal and aromatic plants that are available locally are not investigated yet, and the true estimation of the Emirati EO-bearing plants could be underestimated. Examples of these species include, *Amaranthus graecizans* and *Amaranthus viridis* from the Amaranthaceae family, which were used in the past by the Bedouin people of the UAE to treat scorpion stings, snake bites and itchy skin rashes as reported by Sakkir et al. [28].

**Figure 1:**
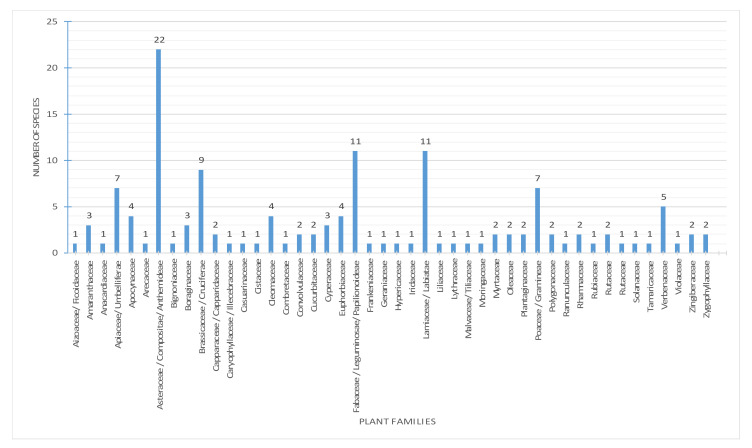
Families of EO-bearing plant species in the UAE.

**Figure 2:**
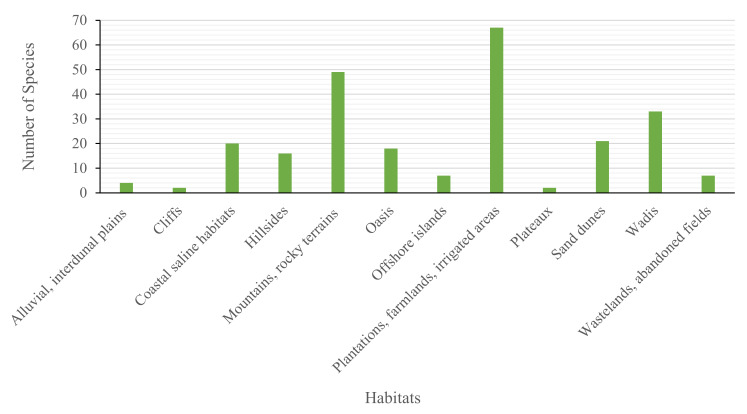
Habitats of EO-bearing plants in the UAE.

**Figure 3:**
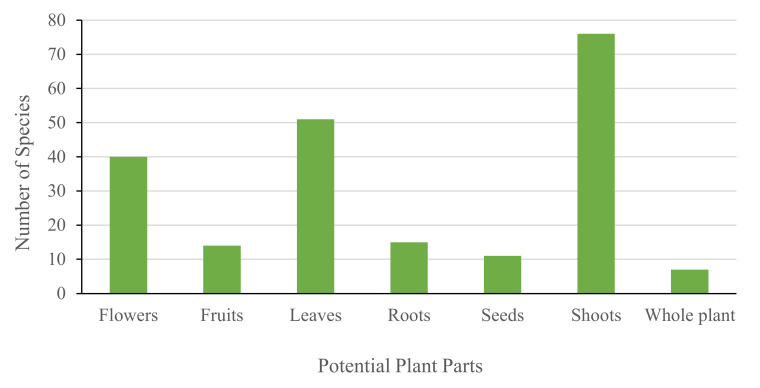
Major plant parts for EO production in the UAE.

**Figure 4:**
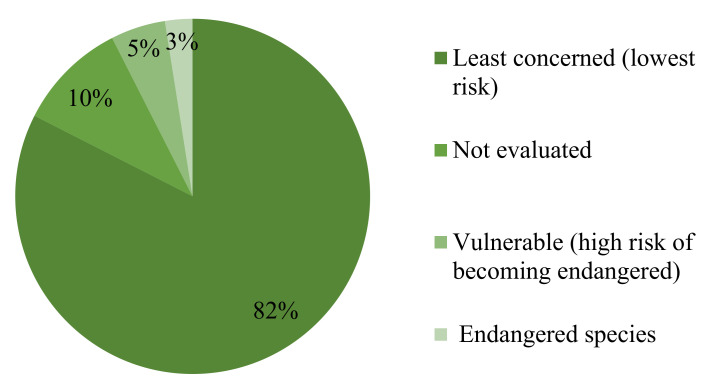
Status of EO-bearing plants in the UAE.

An overview of all EO-bearing plant families growing in the United Arab Emirates, their species number, natural habitats, potential plant parts and ecological status are illustrated in Figure 1, Figure 2, Figure 3 and Figure 4. Based on our results, the families that include the highest number of EO-bearing species are Asteraceae, (22 plants, 16.2%), Fabaceae (11 plants, 8.1%), Lamiaceae (11 plants, 8.1%), Brassicaceae (9 plants, 6.6%), Apiaceae (7 plants, 5.1%) and Poaceae (7 plants, 5.1%), respectively, as shown in Figure 1.

Generally, the most important habitats are the plantations, farmlands and irrigated lands, which host 50% of the Emirati EO-bearing plants. Meanwhile, the most important natural habitats are mountains and rocky terrain, wadis, sandy dunes and coastal saline lines, hosting 36.3, 24.4, 15.5 and 14.8% of the total EO-bearing plants growing in the United Arab Emirates, respectively (Figure 2).

Since plantations and farmlands are hosting 50% of the Emirati EO-bearing plants (which could be due to their ornamental or food production value, or just available naturally as weeds due to the accessibility of water), there should be educational campaigns to educate landlords about extra potentials and economic benefits related to EOs of their available indigenous plants. In addition, with the sharp population growth and the current expansion in the industrial and urbanization activities, strong efforts should be invested to conserve the natural habitats of the EO-bearing plants (e.g., mountains, wadis), and take the same into consideration in strategic planning and management. 

Based on our results, shoots (particularly leaves and flowers) are the most important parts that have potential for EOs, in which, 56.3, 37.8 and 29.6% of the Emirati-EO-bearing plants have the potential to extract EOs from their shoots, leaves and flowers, respectively (Figure 3).

Our results show that 82% of the status of the Emirati EO-bearing plants are reported as least concerned plants (low risks of becoming endangered), as shown in Figure 4. However, since recent references that report the status of indigenous plants are limited, and taking into consideration that status of 10% of the plants are not yet evaluated, their real status could be underestimated, especially with the current population growth and urbanization activities.

### 4.2. A Detailed Overview

A detailed view on the results of the top three richest families (based on the number of EO-bearing species) is divided into three table groups (Table 2, Table 3, Table 4, Table 5, Table 6, Table 7, Table 8, Table 9 and Table 10). The first group represents the general botanical information related to the plant species, including form, life form, life cycle, economic value and folk medicine/applications, internationally and locally (Table 2, Table 5 and Table 8). The second group illustrates the data related to the plants’ natural habitats in the UAE, including important locations, soil, habitats, flowering period and wildlife status (Table 3, Table 6 and Table 9). The third group shows detailed knowledge related to plants’ EOs, including potential plant parts, yields, extraction methods, main chemical groups/constituents and biological activities (Table 4, Table 7 and Table 10).

The results of the top three richest families, based on the number of their species, are illustrated from Table 2, Table 3, Table 4, Table 5, Table 6, Table 7, Table 8, Table 9 and Table 10, including Asteraceae, Fabaceae and Lamiaceae.

The results showed that the taxa of all the UAE EO-bearing plants belongs to the dicotyledon group, except taxa of 15 plants that belong to the monocotyledon group: *Phoenix dactylifera, Cyperus arenarius, Cyperus conglomeratus, Cyperus rotundus, Gynandriris sisyrinchium, Dipcadi erythraeum, Cenchrus ciliaris, Cynodon dactylon, Desmostachya bipinnata, Lolium rigidum, Cymbopogon commutatus, Cymbopogon jwarancusa, Cymbopogon schoenanthus, Alpinia galangal, Zingiber officinale.*

According to our extensive literature review, the Iranian plants show the highest number of publications in the field of EO research. Other important plants’ countries of origin in conducting EOs research include India, Saudi Arabia, Tunisia, Nigeria, Jordan and Pakistan.

On the other hand, there are only a few publications that studied the EO-bearing plants of the UAE. Examples of such studies are mainly by Al Yousuf et al. [7], who studied the EO of *Pulicaria glutinosa* grown in Jebal Al Faya, and Al Yousuf et al., [6] who studied *Teucrium stocksianum* grown in Khor Fakkan. Additional research studied EOs of *Haplophyllum tuberculatum* for plants also grown in Khor Fakkan [5]. Al-Marzouqi et al., [130] studied EO of *Menthe spicata* collected from different regions in the UAE.

Based on the above, there is scarcity in research performed on the EO-bearing plants of the UAE. This is the case while the country has rich biodiversity and has rich traditional medicine applications [16,17,28]. In addition, according to Sakkir et al., [28] 37% of the UAE medicinal plants are applied topically to treat skin problems. This is a direct/indirect indication that the UAE is a good niche for EO phytochemicals of healing benefits. Consequently, it is highly recommended to invest more efforts to study the local EO-bearing plants, seeking new natural resources of phytochemicals of proven biological activity to the country and the world.

Actually, our established databank of the UAE EO-bearing plants offers a solid background to take quick decisions in plant selection and to start up an innovative EOs-based research pathway, which can lead to new chemotypes and promising discoveries. Besides, the databank provides the interested parties (from academic and industrial fields) the opportunity to have an overview on all the Emirati EO-bearing plants, enabling them to highlight the most important indigenous species to supply their needs according to field of interest. At the same time, the databank lists all the UAE EO-bearing plants that need to be conserved from decision-makers to guarantee a sustainable future for the next generation.

It worth mentioning that rich traditional practices are linked (directly or indirectly) to the availability of EOs as active components that lead to particular biological activities of great healing benefits. Additionally, it was reported by a study conducted by Sakkir et al., [28] from the Environmental Agency of Abu Dhabi (EAD) that 37% of the indigenous plants have been used to treat skin problems in the traditional medicine of the UAE. Which can be linked, in one way or another, to the presence of therapeutic grade EO, and therefore could be a positive indicator that the flora of the UAE could pose an excellent resource for EO phytochemicals of various industrial applications.

Thus, it is fundamental to create a comprehensive reference that includes all the UAE indigenous and naturalized species capable of producing EOs. Focusing on the significant role of such a natural resource in a region where fresh water is expensive and where the country’s leadership is working on diversifying the economic resources.

Indeed, it is expected that the essential oil of many indigenous and naturalized medicinal and aromatic plants has not been investigated yet, and the true estimation of the EO-bearing plants growing in the country could be higher than the current findings of this study. Examples of these species include, *Amaranthus graecizans* and *Amaranthus viridis* from the Amaranthaceae family, which were used in the past by Bedouin people of UAE to treat scorpion stings, snake bites and itchy skin rashes as reported by Sakkir et al., [28].

According to published research by Shahin et al., [4], the essential oil of the indigenous medicinal *Cleome amblyocarpa* was extracted and studied for the first time, declaring positive antioxidant activities. Recently, another study extracted essential oil from the seeds of *Moringa peregrina* and evaluated its chemical composition and antioxidant potentials [8]. Therefore, it is expected that similar results can be found while studying other medicinal and aromatic indigenous species.

### 4.3. List of Abbreviations

This section provided the meaning and description of all the abbreviations that were used to construct the tables of the UAE (native and naturalized) EO-bearing plants (Table 2, Table 3, Table 4, Table 5, Table 6, Table 7, Table 8, Table 9 and Table 10).

- Botanical Name: Syn. “Synonyms”; Eng. “English”; Arb. “Arabic”.
- Form: Vine “V”; Grass “G”; Weed “W”; Forb “F”; Herb “H”; Shrub “S”; Tree “T”.
- Life Form: Geophyte “Ge”; Phanerophyte “Ph”; Chamaephyte “Ch”; Hemicryptophyte “He”; Therophyte “Th”; Cryptophyte “Cr”; Neophyte “Na”.
- Life Cycle: Annual “A”; Biennial “B”; Perennial “P”.
- Economic Value: Medicine and/or Folk Medicine “Med”; Food “Fod”; Nutrition “Nutr”; Food Preservation “FPre”; Flavoring “Flav”; Food Aroma “FArom”; Forage “Forg”; Aromatic “Arom”; Essential Oils “EOs”; Cosmetic “Cosm”; Biofuel “Biof”; Fuel “Fuel”; Cleaning and Hygiene “Clea”; Insecticides “Insec”; Ecological “Eco”; Landscaping “Lands”; Other “Oth”: (Dye, Constructions, Household items, Cushions, Fibers, Sponges, Tobacco, Honey Production, Soil Amendment).
- Folk Medicine:

(Application: yes “+”, no “.”) (Country: Applications examples + Plant parts)

Plants’ part abbreviations: Leaves “L”; Stems and twigs “St”; Pods “Pd”; Buds “Bd”; Bark “Bk”; Flowers “Fl”; Shoots and Aerial parts “Sh”; Fruits “Fr”; Seeds “Se”; Whole Plant “W”).

- Emirates: Abu Dhabi “AD”; Dubai “Du”; Sharjah “S”; Fujairah “F”; Ajman “A”; Ras Al Khaimah “RAK”; Umm Al Quwain “UAQ”.
- Important Locations: Al Ain “Ain”; Khor Kalba “K”; Khor Fakkan “KF”; Hajar Mountains “HM”; Ru’us Al-Jibal “RA”; Jebel Hafit “JH”; Wadi Jeema”J”; Hatta “H”; Wadi Lakayyam “WL”; Along the Country “AC”; Country Center “CC”; East Emirates “EE”; North Emirates “NE”; Coasts of North Emirates “CN”; Eastern Coast “EC”; Western coast “WC”; Scattered Locations “SL”
- Soil: Sand “San”; Silt “Sil”; Rocky or Gravel “Roc”; Saline “Sal”.
- Habitats: Oasis “Oas”; Sand Dunes “Dun”; Coasts “Cos”; Roadsides “Rod”; Offshore Islands “Off”; Inland Water Habitats “Wat”; Plantations and Farmlands and Irrigated Lands “Plat”; Hillsides “Hil”; Disturbed Sites “DS”; Alluvial and Interdunal Plains “Apl”; Wadis “Wad”; Gardens “Gar”; Fallow Fields and Plains “FF”; Cliffs “Cli”; Mountains and Rocky terrains “Mou”; Plateaux “PX”; Wastelands and Abandoned Fields “AF”; Urban areas “Urb”.
- Flowering: Months’ abbreviations will be used.
- Wildlife Status: Fairly common and locally abundant “FC”; Common and widespread locally “CO”; Not common “NC”; Rare and vulnerable “RA”; Not evaluated “NE”; Cultivated Plant “C”.
- Plant Part: Potential for EOs (Roots “R”; Rhizomes “Rz”; Tuber “Tu”; Leaves “L”; Stems and twigs “St”; Pods “Pd”; Buds “Bd”; Bulbs “Bl”; Bark “Bk”; Flowers “Fl”; inflorescences “IF”; Shoots and Aerial parts “Sh”; Fruits “Fr”; Seeds “Se”; Whole Plant “W”).
- Physical Properties: EOs physical characteristics (Color/Odor) Plant part + extraction method.
- Yield (%): EO yield (%, *v*/*w* of dry weight). Supported with plant part and extraction method
- Isolation Method: EO extraction method including:

Hydrodistillation “HD”; Steam Distillation “SD”; Dry Steam Distillation “DSD”; Microdistillation “MD”; Solid-Phase Microextraction “SPM”; Simultaneous Steam Distillation and Extraction “SDE”; Vacuum Distillation “VD”; Ligarine Extraction “LE”; Soxhelt Extraction “SH”; Headspace Analysis “H”; Gas Chromatography Flame Ionization Detector “GC-FID”; Supercritical CO2 Fluid Extraction “SFE”; Microwave-Assisted Hydrodistillation “MW”; Solvent-Free Microwave Extraction “SFME”; Tenax-Trapping “TT”.

- Main Chemical Groups/Components:

(Main EOs Chemical Groups) and/or (Main/Potential Chemical Constituents) Plant part + extraction method.

- Biological Activity: [EO Biological Activity] (Activity Details) Plant part + extraction method.

Antitumor “AT”; Antioxidant “AO”; Antifungal “AF”; Antibacterial “AB”; Antimicrobial “AM”; Antibiotic “OT”; Anti-inflammatory “AI”; Antianxiety “AA”; Mosquito Attractant/Repellent “MR”; Insecticidal and Pesticidal Activity “IS”; Larvicidal Activity “LA”; Nematicidal activity “NM”; Oviposition attractant/deterrent activity “OA”; Antihelminthic “Anthelmintic” effect “AH”; Antiechinococcal Activity “AE”; Fumigant Toxicity “FT”; Antidiabetic Activity “AD”; Antistreptococcal “AS”; Anticarcinogenic Effect “AC”; Cytotoxic Properties “CP”; Antimycotoxins “XN”; Phytotoxic Properties and Herbicidal Activity “PP”; Apoptotic Properties “AP”; Antimutagenic Properties “MP”; Analgesic properties “GP”; Antidepressant “DP”; Antispasmodics properties “ASP”; Antinociceptive activity “AN”; Antinociceptive Activity “CE”; Antiseptic “SP”; Stimulant “ST”; Antidiarrheal Activity “DR”.

- General Notes: The use of “!” means information uncertainty.

### 4.4. Phytochemicals and Biological Activities from UAE Based Plants

Reviewing the literature, essential oils of the following six native/naturalized UAE plants were investigated under UAE climatic conditions, including *Pulicaria glutinosa* (Asteraceae) [7], *Cleome amblyocarpa* (Cleomaceae) [4], *Mentha spicata* (Lamiaceae) [130], *Teucrium stocksianum* (Lamiaceae) [6], *Haplophyllum tuberculatum* (Rutaceae) [5] and *Moringa peregrine* (Moringaceae) [8], with rich therapeutic applications for the last five species in folk medicine generally and the UAE traditional practices specifically. For example, infusion of *C. amblyocarpa* leaves was used to treat abdominal and rheumatic pain. *M. spicata* was used to promote general health-care benefits. Meanwhile, *T. stocksianum* has various applications related to kidney, stomach pains, thyroid problems and the common cold. The leaves of *H. tuberculatum* were used to treat scorpion stings, eaten as sedative and crushed in water and drunk to treat painful joints. In the UAE folk practices, the seeds’ oil of *M. peregrine* has been taken orally for constipation and stomach cramp, and the seeds’ oil mixture with clove oil and cardamom oil has been taken as a drink during labor. Besides, the seeds’ oily extract is used to treat headaches, fever, muscle pains, burns, abdominal pain and constipation. *M. peregrine* leaves’ extract can be rubbed on skin to treat a skin rash [22].

The rocky soils of the Hajar mountain are among the most famous places for *P. glutinosa*, *T. stocksianum, H. tuberculatum* and *M. peregrine,* including Khor Fakkan and Ru’us Al-Jibal for *T. stocksianum* and *M. peregrine.* While the sandy soils of the North Emirates of Dubai, Sharjah, Ajman and Umm Al Quwain are rich in *C. amblyocarpa*, the mountains, hillsides and wadis of Fujairah, Ras Al Khaimah, Sharjah and Abu Dhabi are rich places for *H. tuberculatum* [21]. The mint herb *M. spicata* is widely cultivated in farms for food production purposes, and contributes to the richest essential oils yield which is 10.90%, extracted from shoots using Supercritical carbon dioxide (SCCO_2_) (Press: 350 bar, Temp: 50°C) [130]. While the essential oil average yields of the aerial parts of each of *P. glutinosa, T. stocksianum*, *H. tuberculatum* and *C. amblyocarpa*, were (according to the highest yield reported in the literature based on UAE) 0.5 [7], 0.34 [6], 0.04 [5] and 0.0266% [4], respectively. The essential oil seeds’ oil of *M. peregrine* extracted by hydrodistillation reported to be 0.22% [8].

A study of the phytochemicals showed that the major constituents of *P. glutinosa* essential oils extracted from aerial parts (including flowers) by steam distillation were *p*-elemene, 7-cadinol and *a*-cadinol (Sesquiterpenes) [7]. No studies were found to test the biological activities of the essential oil extracts for this shrub.

According to Shahin et al., [4], the major phytochemicals found for *C. amblyocarpa* essential oil (extracted by hydrodistillation from the whole herb) were isobornyl formate, tetrahydro-linalool acetate, neo-menthyl acetate, 1-dodecene and *γ*-elemene. The extract showed antioxidant activities (in vitro) using DPPH, FRAP and ABTS assays.

As reported by Al-Marzouqi et al., [130], the main chemical composition of *M. spicata* leaves’ essential oil (extracted by SCCO_2_) included carvone, *a*-pinene, limonene and linalool, which were significantly higher in the locally cultivated *M. spicata* in comparison to herbs imported from France, Syria and India. Although many studies in the literature reported the various biological activities of *M. spicata* (e.g., antibacterial, antifungal, antimicrobial, antioxidant, insecticidal and pesticidal, larvicidal activity, mosquito attractant/repellent and antimutagenic properties), however, no studies thus far have tested the biological activity of the oil for the herb cultivated under UAE climatic conditions.

According to Al Yousuf et al. [6], the oil of the aerial parts of *T. stocksianum,* collected from the UAE, was characterized by *a*-cadinol and 6-cadinene. Studies based on other countries reported the antinociceptive activity of the oil, with no studies found related to the biological activity of the oil based on the UAE.

Based on the research findings of Al Yousuf et al., [5], the oil extracted from the aerial parts of *H. tuberculatum α*-phellandrene (10.7–32.9%) being the major component and with significant amounts of other phytochemicals varied in existence and percentages according to the harvesting season. Such phytochemicals include *β*-caryophyllene, *β*-pinene, limonene, *δ*-3-carene, linalool, linalyl acetate, *β*-caryophyllene and *α*-terpineol. The biological studies related to *H. tuberculatum* carried out based on other countries reported that the oil exhibits various biological activities including antifungal, antibacterial, antimicrobial, mosquito attractant/repellent, insecticidal and pesticidal activity and larvicidal activity. On the other hand, no studies have been conducted yet to test these activities and others for the essential oil of this perennial herb grown under the UAE climate.

According to Senthilkumar et al. [8], the seeds’ oil of *M. peregrine* was characterized by the availability of geijerene (33.38%), linalool (23.36%), caryophyllene oxide (19.28%), n-hexadecane (12.59%) and carvacrol. The oil was found to be a potential alternative choice to the synthetic antioxidants, having radical scavenging activities including; DPPH^•^ radical (IC_50_ = 37.70 μg/mL), ABTS^•+^ radical (IC_50_ = 34.03 μg/mL), superoxide anion (IC_50_ = 36.57 μg/mL), nitric oxide radical (IC_50_ = 29.15 μg/mL), hydrogen peroxide (IC_50_ = 43.93 μg/mL) and hydroxyl radical (IC_50_ = 29.99 μg/mL).

Studies of phytochemicals and biological studies provide scientific justification for the rich therapeutic applications of the previously mentioned native/naturalized plants in the UAE traditional practices. At the same time, it is obvious that there is a lack of essential oil studies based on the UAE, and more efforts are needed to investigate the phytochemicals and biological activities of oils extracted from locally grown and harvested native plants. Besides, comparative studies to compare the essential oil yield (quantitatively and qualitatively) for plants grown in the UAE and other countries are required. This is needed to highlight the native/naturalized plants of superior quality and biological activity, and utilize the same (after standardization) for commercial purposes in various industries (e.g., pharmaceuticals, cosmetics, food preservatives, fragrance and flavor industries).

## 5. Obstacles and Difficulties

The greatest obstacles and difficulties that were faced are related to the scarcity of the references to UAE wildflowers (Shahin, 2018c). There is so much confusion in the literature between the botanical names and the synonyms, including spelling mistakes that make the task of data collection to list all the Emirati plants (followed by screening and listing the Emirati EO-bearing plants) a difficult and complicated mission.

For example, *Cornulaca arabica* Botsch and *Cornulaca monacantha* Delile were mentioned as two different species in the reference of Brown and Sakkir [22], while according to published study [21] *Cornulaca arabica* Botsch is a synonym of *Cornulaca monacantha* Delile. Besides, the plants *Actiniopteris semiflabellata, Commicarpus boissieri* and *Cymbopogon jwarancusa* were mentioned with minor spelling mistakes as *Actioniopteris semiflabellata, Commicarpus boisieri* and *Cymbopogon jwarancuse,* (respectively) in the textbook of Jongbloed et al., [21] which is one of the most important references of the UAE indigenous and naturalized plants.

Moreover, some publications use either the synonyms or the common names instead of using the botanical names. Therefore, while reviewing the literature using the formal botanical names (to screen the EO potential) no results will appear, although, in many cases the plant would be a rich resource of EO phytochemicals. For example, some publications will use *Dipcadi serotinum, Cymbopogon parkeri* Stapf. *Heliotropium europaeum* and *Calligonum polygonoides* instead of using the botanical names, which are *Dipcadi erythraeum* Webb and Berth., *Cymbopogon commutatus* (Steud.) Stapf., *Heliotropium lasiocarpum* and *Calligonum comosum*, respectively.

## 6. Conclusions and Future Perspectives

Based on our comprehensive and detailed screening of all the families of the UAE wildflowers, we concluded that there are at least 137 EO-bearing plants in the UAE (17% of the UAE wildflowers) belonging to 46 families. The top three richest families, based on the number of their species, are Asteraceae, Fabaceae and Lamiaceae.

Most of the UAE EO-bearing plants have rich traditional medicinal applications and other economic values, such as pharmaceuticals, nutrition, aromatherapy, fragrance and flavoring. Generally, the shoots (especially leaves and flowers) are the most important parts to extract EO phytochemicals (e.g., terpenoids) of valuable biological activities, such as antioxidant, antimicrobial and antitumor properties.

The UAE EO-bearing plants are widespread in the areas of plantations, mountains and wadis of the country. Serious efforts to educate landlords about the great value of the UAE EO-bearing plants are needed, to make sure that these expensive species are well-cultivated in a sustainable manner. Besides, strong efforts related to management and strategic planning should be employed to conserve the natural habitats of the EO-bearing plants.

All our obtained results support that the UAE is a rich natural resource for the native and naturalized EO-bearing plants that have rich ethnobotanical applications of multiple economic potential.

Therefore, serious efforts are needed to standardize the oil yield (quantitatively and qualitatively) for all listed essential oil-bearing plants of the UAE, and to focus sustainability on native essential oil-bearing plants of industrial applications at research and commercialization level. Taking into consideration that this field is promising for multiple research disciplines and discoveries.

## Figures and Tables

**Table 1:** An overview of indigenous and naturalized essential oil-bearing plants of the UAE.

	Family	Binomial	Taxonomic Authority	Synonyms “Syn.” and/or Common Names (English “Eng.” and/or Arabic “Arb.”)	Reference Categorizing the Plant as Essential Oil-Bearing Plant	Reference for UAE Nativity/Natuaralization
1	Aizoaceae/Ficoidaceae (Mesembryanthemum, carpetweed family)	*Sesuvium portulacastrum*	L.	*Sesuvium verrucosum* Raf. (Eng. Shoreline purslane, sea purslane, sesuvium)	[35]	[21,22]
2	Amaranthaceae (Cockscomb family)	*Achyranthes aspera*	L.	(Eng. Prickly chaff flower) (Arb. Saif el-jinn, umdhrese, sehem, ar-ray, mahoot, na’eem, wazer)	[36]	[21,22]
3		*Aerva javanica*	(Burm. f.) Juss. ex Schul.	(Eng. Desert cotton, snow bush) (Arb. Al ara’, twaim, efhe, tirf)	[37,38]	[15,18,21,22,23,24,25,27,28,32]
4		*Chenopodium album*	L.	(Eng. Lamb’s quarters, melde, goosefoot, fat-hen, white goosefoot) (Arb. Shulah, ‘aifajan, rokab al-jamal)	[39]	[15,21,22]
5	Anacardiaceae (The cashew, sumac family)	*Pistacia khinjuk*	Stocks.		[40]	[21]
6	Apiaceae/Umbelliferae	*Ammi majus*	L.	(Eng. Bishop’s flower, bishop’s weed) (Arb. Sannout, sheeh, khilla “khilla sheitani”, nayniya)	[41]	[15,21,22,28]
7		*Anethum graveolens*	L.	(Eng. Dill Weed)	[42]	[15,21]
8		*Ducrosia anethifolia*	(DC.) Boiss.	(Arb. Basbaz, haza)	[43]	[15,21,22,28]
9		*Pimpinella eriocarpa*	Banks and Sol.	(Arb. Kusaybirah)	[44]	[15,21]
10		*Pimpinella puberula*	(DC.) Boiss.		[45]	[15,21]
11		*Scandix pecten-veneris*	L.	(Eng. Shepherd’s-needle, Crib Gwener) (Arb. Mushet)	[46]	[15,21]
12		*Torilis leptophylla*	(L.) Reichenb.f.	(Syn. *Caucalis leptophylla*) (Eng. Bristle-fruited hedge-parsley)	[47]	[21]
13	Apocynaceae (Dogbane family)	*Catharanthus roseus*	(L.) G. Don	(Syn. *Vinca rosea*) (Eng. Madagascar periwinkle)	[42]	[15]
14		*Nerium oleander*	L.	(Syn. *Nerium mascatense*) (Eng. Rosebay, olender) (Arb. Defla, haban)	[48]	[15,21,22,28]
15		*Plumeria rubra*	L.	(Eng. Nosegay, frangipan)	[49]	[15,21,22,28]
16		*Calotropis procera*	(Aiton) W.T. Aiton	(Eng. Apple of Sodom, Sodom apple, stabragh, kapok tree, king’s crown, rubber bush, rubber tree, Sodom’s apple milkweed) (Arb. ‘ushar, shakjr, ‘asur, ashkhar “askar”)	[50]	[21,22,28]
17	Arecaceae (Palmae, palmaceae family, palm trees)	*Phoenix dactylifera*	L.	(Eng. Date palm, date palm tree) (Arb. Nakhl, amm-amm)	[51]	[15,21,22,28]
18	Asteraceae/Compositae/Anthemideae (Sunflower family)	*Anthemis odontostephana*	Boiss.	(Arb. O’qhowan)	[52]	[15,21]
19		*Artemisia sieberi*	Besser	(Eng. Wormwood)	[53]	[28]
20		*Calendula arvensis*	L.	(Eng. Field marigold) (Arb. Ain el baqr, eqhwan-asfar, hanwa, hanuwa)	[54]	[15,21]
21		*Cichorium intybus*	L.	(Eng. Blue daisy, blue dandelion, blue sailors, blue weed, bunk, coffeeweed) (Arb. hindibaa bareeya, chicoria)	[55]	[15,21,28]
22		*Conyza bonariensis*	(L.) Cronq.	(Syn. *Conyza linifolia* (Willd.) Tackh) (Eng. Flax-leaf Fleabane, Wavy-leaf Fleabane, hairy fleabane) (Arb. hashishat el-jebal, tebaq)	[56]	[21,22]
23		*Eclipta prostrata*	L.	(Syn. *Eclipta alba* (L.) Hassk.) (Eng. False daisy, Trailing eclipta ). (Arb. Sa’ada, sauweid, masadate)	[57]	[15,21]
24		*Grantia aucheri*	Boiss.	No Information	[58]	[27]
25		*Launaea nudicaulis*	(L.) Hook. f.	(Eng. Hawwa Baqrah ara, hindabah ara, huwah ara, naked launea) (Arb. Huwah al ghazal, safara, huwah, hindabah)	[59,60]	[15,21,22]
26		*Matricaria aurea*	(Loefl.) Sch. Bip.	(Eng. Golden chamomile) (Arb. Babunaj)	[61]	[21,28]
27		*Matricaria chamomilla*	L.	(Syn. *Matricaria recutita*) (Eng. Chamomile, camomile, german chamomile)	[62]	[63]
28		*Pluchea arabica*	(Boiss.) Qaiser and Lack	(Eng. Pluchea) (Arb. godot)	[64]	[28]
29		*Pluchea dioscoridis*	(L.) DC.	(Syn. *Conyza dioscoridis* (L.) Desf., *Baccharis dioscoridis* L.) (Eng. Ploughmans spikenard, marsh fleabane) (Arb. Sahikee, barnof)	[65]	[15,21,22]
30		*Pluchea ovalis*	(pers.) DC.	(Eng. Woolly camphor-weed)	[66]	[15,21]
31		*Pseudognaphalium luteo-album*	(L.) H. and B.	(Syn. *Gnaphalium luteo-album* L.) (Eng. Cudweed) (Arb. sabount el’afrit)	[67]	[21,22]
32		*Pulicaria arabica*	(L.) Cass.	(Syn. *Inula arabica* L./*Pulicaria elata* Boiss./*Pulicaria laniceps* Bornm.) (Arb. Iqat, abu ‘ain safra)	[68]	[21]
33		*Pulicaria glutinosa*	Jaub. and Spach	(Arb. Thal, fal, shajarat fal, muhayda, mithidi, shajarat al-mithidi, shneena, zayyan)	[7]	[15,21,22]
34		*Pulicaria inuloides*	(Poir.) DC.	No information	[69,70,71]	[21]
35		*Pulicaria undulata*	(L.) C.A. Meyer	(Syn. *Pulicaria crispa* (Forssk.) Benth.) (Eng. Crisp-leaved fleabane) (Arb. Gathgeth, jithjath, ‘urayfijan)	[72]	[15,21,22]
36		*Rhanterium epapposum*	Oliv.	(Eng. Rhanterium) (Arb. Arfaj)	[73]	[15,21,22]
37		*Senecio glaucus*	L. ssp. coronopifolius (Maire) Al.	(Syn. *Senecio desfontanei* Druce) (Eng. Maire alexander, buck’s horn groundsel) (Arb. Qorreis, murair, zamlooq, shakhees, rejel al ghurab)	[74]	[15,21,22]
38		*Seriphidium herba-alba*	(Asso) Sojak	(Syn. *Artemisia herba-alba* Asso/Artemisia inculta Del) (Eng. Wormwood, white wormwood) (Arb. Ata, ghata, shih)	[75,76]	[21]
39		*Sphagneticola trilobata*		(Syn. *Wedelia paludosa* DC.) (Eng. Singapore daisy, creeping-oxeye, trailing daisy, wedelia)	[77]	[15]
40	Bignoniaceae (Bignonias family)	*Jacaranda mimosifolia*	D. Con	(Eng. Jacaranda, blue jacaranda, black poui, fern tree)	[78]	[15]
41	Boraginaceae (Borage, forget-me-not family)	*Arnebia linearifolia*	DC.	No Information	[79]	[21]
42		*Heliotropium europaeum*	L.	(Syn. *Heliotropium lasiocarpum* Fisch. and Mey.) (Eng. European heliotrope, european turnsole) (Arb. Karee)	[80]	[15,21,22]
43		*Trichodesma africanum*		(Syn. *Trichosdesma africana* (L.) R. Br.) (Eng. African barbbell)	[81]	[15]
44	Brassicaceae/Cruciferae (Cress, mustard family)	*Capsella bursa-pastoris*	(L.) Medik.	(Eng. Shepherd’s-purse) (Arb. Kis al ra’y)	[82]	[15,21,22]
45		*Cardaria draba*	(L.) Desv.	(Syn. *Lepidium draba*) (Eng. Whitetop, hoary cress) (Arb. lislis)	[83]	[21,22]
46		*Coronopus didymus*	(L.) Sm.	(Eng. Lesser swine-cress) (Arb. Rashad al-barr)	[84]	[21,22]
47		*Erucaria hispanica*		(Eng. Spanish pink mustard, erucaria myagroides) (Arb. Khezaam, saleeh, kromb al sahra)	[79]	[15,21]
48		*Eruca sativa*	Mill.	(Eng. Salad rocket, rucola, rucoli, rugula, colewort, roquette, garden rocket, rocket) (Arb. Girgir, jirjeer)	[85]	[15,21,22,28]
49		*Savignya parviflora*	(Delile) Webb	(Eng. Jaljalan, kanad al barr, gulgulan, girgees, small whorled cheeseweed) (Arb. Khzaymah, al-thee, jerjees “girgees”, gongolan “qunqulan, jaljelan, galeigelan, bithman”)	[86]	[15,21,22]
50		*Schimpera arabica*	Hochst. and Steud.		[79]	[15,21]
51		*Sinapsis arvensis*	L.	(Syn. *Sinapis arvensis* L.) (Eng. Charlock, charlock mustard, wild mustard)	[87]	[15,21]
52		*Sisymbrium irio*	L.	(Eng. London rocket) (Arb. Howairah, shelyat, figl el-gamal, harrah) قراص حمار	[88]	[21,28]
53	Capparaceae/Capparidaceae (Caper family)	*Capparis cartilaginea*	Decne.	(Eng. Caper) (Arb. Qubr, sediru, ashflah, lezaf “losef, lusfeh, ewsawf)	[89]	[15,21,22,26,28]
54		*Capparis spinosa*	L.	(Eng. Caper bush, flinders rose) (Arb. Kobar, lasafa, fakouha, shawk mal homar, shafallah, delayeer, dabayee)	[89,90]	[15,21,22,28]
55	Caryophyllaceae/Illecebraceae (Carnation family)	*Stellaria media*	(L.) Vill.	(Eng. Chickweed, common chickweed, chickenwort, craches, maruns, winterweed, lawn weed) (Arb. Meshit, hashishet el-qizaz, qizaza)	[91]	[15,21]
56	Casuarinaceae (Beefwood family)	*Casuarina equisetifolia*		(Syn. *Casuarina equistetifolia* J.R. and G. Forst.) (Eng. Rhu, casuarina tree)	[92,93]	[15]
57	Cistaceae (Rock-rose, rock rose family)	*Helianthemum kahiricum*	Delile	(Eng. Rock rose, sun rose) (Arb. Ragroog, qsasah, hashma)	[94]	[15,21,22]
58	Cleomaceae	*Cleome amblyocarpa*	Barr. and Murb.	(Syn. *Cleome africana*, *Cleome arabica*, *Cleome daryoushian*) (Eng. Spider flower) (Arb. Adheer, durrayt an-na’am, khunnayz, ufaynah)	[4,95]	[21]
59		*Cleome brachycarpa*	Vahl ex DC.	(Syn. *Cleome vahliana* Farsen) (Arb. Za’af, mkhaysah)	[96]	[15,21]
60		*Cleome droserifolia*	Del.	(Syn. *Roridula droserifolia* Forssk.) (Eng. Cleome herb)	[97]	[15]
61		*Cleome gynandra*	L.	(Syn. *Gynandropsis gynandra* (L.)Briq.) (Eng. Shona cabbage, African cabbage) (Arb. Abu qarim)	[98]	[21]
62	Combretaceae	*Terminalia catappa*	L.	(Eng. Indian almond-wood, bastard almond, andaman badam) هليلج هندي	[99]	[15]
63	Convolvulaceae (Morning glory, bindweed family)	*Ipomoea aquatica*	Forssk.	(Eng. Kang kong, water convolvulus, water spinach, swamp cabbage, ong choy, hung tsai, rau muong) السبانخ المائي	[100]	[21,22]
64		*Ipomoea obscura*		(Eng. Obscure morning-glory, small white morning glory)	[101,102]	[21]
65	Cucurbitaceae (Gourd family)	*Momordica charantia*	L.	(Eng. Bitter melon, bitter gourd, bitter squash, balsam-pear)	[103,104]	[24]
66		*Luffa acutangula*	(L.) Roxb.	(Eng. Angled luffa, chinese okra, dish cloth gourd, ridged gourd, sponge gourd, vegetable gourd, strainer vine, ribbed loofah, silky gourd, ridged gourd, silk gourd, sinkwa towelsponge)	[103,105]	[21]
67	Cyperaceae (Sedges family)	*Cyperus arenarius*	Retz.	(Syn. *Bobartia indica* L.) (Eng. dwarf sedge)	[106]	[21,22,34]
68		*Cyperus conglomeratus*	Rottb.	(Eng. Cyperus, mali tamachek saad) (Arb. Thenda. Ayzm, chadrum, qassis, rasha)	[107]	[15,21,22,34]
69		*Cyperus rotundus*	L.	(Eng. Coco-grass, Java grass, nut grass, purple nut sedge, red nut sedge, Khmer kravanh chruk) (Arb. Sa’ed, sa’ed al hammar, hasir)	[108]	[15,21,22,28,34]
70	Euphorbiaceae (Spurge, castor, euphorbias family)	*Euphorbia helioscopia*	L.	(Eng. Sun spurge, madwoman’s milk) (Arb. Haleeb al-diba, sa’asa, tanahout, kerbaboosh)	[109]	[21,22]
71		*Euphorbia hirta*	L.	(Eng. Asthma plant, asthma weed, pill-bearing spurge) (Arb. Libbein, demeema, menthra)	[110]	[15,21,22]
72		*Euphorbia peplus*	L.	(Syn. *Euphorbia peplis* L.) (Eng. Petty spurge, radium weed, cancer weed, milkweed) (Arb. Khunaiz)	[111]	[21,22,28]
73		*Ricinus communis*	L.	(Eng. Castor oil) (Arb. ‘Arash, ash’asheh, khasaab, khirwa “khurwa’a”, junijund, tifsh)	[112]	[15,21,22,28]
74	Fabaceae/Leguminosae/Papilionoideae (Pea family)	*Alhagi maurorum*	Medik.	(Syn. *Alhagi graecorum* Boiss.) (Eng. Camelthorn, camelthorn-bush, caspian manna, persian mannaplant) (Arb. Shwaika, agool, heidj)	[113,114]	[15,24]
75		*Lotus halophilus*	Boiss. and Spruner	(Eng. Greater bird’s foot trefoi) (Arb. Horbeith “hurbuth”, garn al ghazal, ‘asheb al ghanem)	[79,89]	[15,21,22]
76		*Medicago polymorpha*	L.	(Eng. California burclover, toothed bur clover, toothed medick, burr medic)	[115]	[15,21,22]
77		*Medicago sativa*	L.	(Eng. Alfalfa, lucerne)	[116]	[15]
78		*Rhynchosia minima*	(L.) DC.	(Eng. least snout-bean, burn-mouth-vine and jumby bean) (Arb. Baql)	[117]	[15,21,22]
79		*Tephrosia persica*	Boiss.	(Syn. *Tephrosia apollinea* (Delile) DC.) (Arb. Dhafra, omayye, nafal)	[118]	[15,21,32]
80		*Trigonella hamosa*	L.	(Eng. Branched fenugreek, Egyptian fenugreek) (Arb. Nafal, qutifa, qirqas, darjal, eshb al-malik, qurt)	[79]	[15,21,22]
81		*Ononis sicula*	Guss.	------	[119]	[21]
82		*Acacia nilotica*	(L.) Delile	(Syn. *Acacia Arabica* (Lam.) Willd.) (Eng. Gum arabic tree, babul/kikar, Egyptian thorn, sant tree, al-sant, prickly acacia) (Arb. Sunt garath “kurut”, babul, tulh. Fruit: karat)	[120,121]	[15,21,22,26,28]
83		*Acacia tortilis*	(Forssk.) Hayne	(Eng. Umbrella thorn) (Arb. Samr “samur”, salam)	[120,122]	[15,21,22,26,32]
84		*Prosopis farcta*	(Banks and Sol.) Mac.	(Eng. Dwarf mesquite, syrian mesquite) (Arb. Yanbut, agoul, awsaj)	[123]	[15,21,22]
85	Frankeniaceae	*Frankenia pulverulenta*	L.	(Eng. European Frankenia, European sea heath) (Arb. Molleih, hamra (hmaira), Umm thurayb)	[124]	[21,22]
86	Geraniaceae (Geranium family)	*Erodium cicutarium*	(L.) L Her. Ex Aiton	(Eng. Redstem filaree, redstem stork’s bill, common stork’s-bill, pinweed)	[125]	[21]
87	Hypericaceae (St. Johnswort family)	*Hypericum perforatum*		(Eng. St.John’s wort)	[126]	[30]
88	Iridaceae (Irises family)	*Gynandriris sisyrinchium*	(L.) Parl.	(Syn. *Iris sisyrinchium* L., *Moraea sisyrinchium* (L.) Ker Gawl.) (Eng. Barbary Nut, mountain iris) (Arb. Khowais, su’ayd, ‘unsayl)	[127]	[15,21]
89	Lamiaceae/Labiatae (Mint, deadnettle family)	*Lallemantia royleana*	(Benth.) Benth.	(Eng. Bian bing cao)	[128]	[21]
90		*Mentha spicata*		spicata (Eng. Spearmint, spear mint)	[129]	[63,130]
91		*Ocimum forsskaolii*	Benth.	(Syn. *Ocimum forskolei* Benth.) (Eng. Rehan, sawma) (Arb. Basil)	[131]	[15,21,28]
92		*Salvia aegyptiaca*	L.	(Eng. Egyptian sage) (Arb. Ra’al, na’aim, ghbeisha, shajarat al ghazal, khizam)	[132]	[15,21,22,24,28]
93		*Salvia macilenta*	Boiss.	(Eng. Khizama) (Arb. Khmayzah lethnay, bithman)	[133]	[15,21,22]
94		*Salvia macrosiphon*	Boiss.	(Arb. Shajarat Al Ghazal)	[134]	[15,21]
95		*Salvia mirzayanii*	Rech.f. and Esfandiari		[135]	[21]
96		*Salvia spinosa*	L.	(Arb. Shajarat al-ghazal)	[136]	[21,22]
97		*Teucrium polium*	L.	(Eng. Felty germander)	[137,138]	[21,28]
98		*Teucrium stocksianum*	Boiss.	(Eng. Jadah, yadah, Ja‘adah) (Arb. Ya’dah, brair)	[6,139]	[15,21,22,24,32,28]
99		*Zataria multiflora*	Boiss.	(Eng. Za’atar, shirazi thyme)	[140,141]	[24]
100	Liliaceae (lily family)	*Dipcadi erythraeum*	Webb and Berth.	(Synonym: *Dipcadi serotinum* (L.) Medik.) (Eng. Brown Lily, Hyacinthus serotinus, mesailemo, besailemo) (Arb. Busalamo, ansel, misselmo, shkal).	[142]	[15,21,22]
101	Lythraceae	*Lawsonia inermis*	L.	(Eng. Egyptian Privet, the henna tree, mignonette tree)	[143]	[15,21,22,24,28]
102	Malvaceae/Tiliaceae (Mallows family)	*Corchorus depressus*	(L.) Stocks.	(Eng. Mulakhiyah al bar, sutaih, rukbat al jamal) (Arb. Matara, seluntah, mulukhia el bar, waikai)	[144]	[15,21,22]
103	Moringaceae (Moringa family)	*Moringa peregrina*	*(Forssk.) Fiori*	(Eng.Wild drumstick tree) (Arb. Shu’, yasar, baan, ’aweyr, bayreh, terfaal, yayn)	[8]	[21]
104	Myrtaceae (Myrtle family)	*Eucalyptus camaldulensis*	Dehnh.	(Syn. *Eucalyptus camaldulensis* Dehn.) (Eng. River red gum, red gum, Murray red)	[145]	[15]
105		*Eucalyptus pimpiniana*	Maiden	“Eng. Pimpin mallee, red dune mallee”	[146]	[15]
106	Oleaceae (Olive family)	*Jasminum sambac*	(L.) Ait.	(Eng. Arabian jasmine) الفل	[147]	[15]
107		*Olea europaea*	L. subsp. Cuspidata	(Wall. Ex G. Don) ciferri (Eng. Olive tree) (Arb. ‘Itm, mitan)	[148]	[21]
108	Plantaginaceae (Plantain family)	*Plantago amplexicaulis*	Cav.	(Eng. Ispaghula, Plantain, rablat al mistah, lesan al hamal) (Arb. gerenwa, rabl, aynsum, khannanit an na’ja)	[79]	[15,21,22]
109		*Plantago boissieri*	Hausskn. and Bornm.	(Arb. Rabl, yanam)	[149]	[15,21,22]
110	Poaceae/Gramineae (Gramineae, true grasses family)	*Cenchrus ciliaris*	L.	(Eng. Buffelgrass, African foxtail grass, sand-burr) (Arb. Sadat, khadir, thumum, gharaz, drab, labaytad)	[79]	[15,21,32,34]
111		*Cynodon dactylon*	(L.) Pers.	(Eng. Bermudagrass, dubo, dog’s tooth grass, Bahama grass, devil’s grass, couch grass) (Arb. Thi’il, negil “najiel”, najm, sheel, bizait)	[150,151]	[15,21,34]
112		*Desmostachya bipinnata*	(L.) Stapf	(Eng. Halfa grass, big cordgrass, salt reedgrass) (Arb. Halfa, hafla and sanaiba)	[152]	[15,21,34]
113		*Lolium rigidum*	Gaudin	(Eng. Wimmera ryegrass, Swiss rye grass) (Arb. Hayyaban, shilm, ziwan, simbil, rabiya)	[153]	[15,21]
114		*Cymbopogon commutatus*	(Steud.) Stapf	(Syn. *Cymbopogon parkeri* Stapf.) (Eng. Incense grass, Rosagrass) (Arb. Alklathgar, sakhbar, hamra, idhkhir, khasaab)	[154]	[15,21,22,34]
115		*Cymbopogon jwarancusa*	subsp. olivieri (Boiss.)	(Eng. Oilgrass, iwarancusa grass)	[155]	[20,21]
116		*Cymbopogon schoenanthus*	(L.) Spreng.	(Eng. Camel grass, camel’s hay, fever grass, geranium grass, West Indian lemon grass) (Arb. Adlghar, hashmah)	[155,156]	[15,21,22,24,28,34]
117	Polygonaceae (buckwheat family)	*Calligonum comosum*	L’Her.	(Synonym: *Calligonum polygonoides* subsp. comosum (L‘Her.) Soskov) (Eng. Fire bush) (Arb. Arta, waragaat as-shams, ‘abal, dhakar)	[157,158,159]	[15,21,22,24,28]
118		*Rumex vesicarius*	L.	(Eng. Sorrel, Bladder dock, Rosy dock, Ruby dock) (Arb. Humayth “hommeid, hummad, hambad”, hambasees)	[160]	[15,21,22,24,28]
119	Ranunculaceae (Buttercup, crowfoot family)	*Nigella sativa*	L.	(Eng. Black seed, black cumin)	[161]	[24]
120	Rhamnaceae (Buckthorn Family)	*Ziziphus jujuba*	Mill.	(Eng. Chinese date, jujube)	[162]	[15]
121		*Ziziphus spina-christi*	(L.) Willd.	(Eng. Christ’s thorn jujube, Christ’s torn, nabk tree) (Arb. Sidr, ber, ‘ilb, zaqa)	[163]	[15,21,22,28]
122	Rubiaceae (coffee, Rue, madder, bedstraw family)	*Galium tricornutum*	Dandy	(Eng. Rough corn bedstraw, roughfruit corn bedstraw and corn cleavers)	[164]	[21]
123	Rutaceae (Rue, citrus family)	*Haplophyllum tuberculatum*	(For.) A. Juss.	(Syn. *Haplophyllum arabicum*) (Eng. Sazab, zeita, kheisa, mesaika) (Arb. Srayu’u asraw, mekhiseh “Umm musayka”, kirkhan, zuqayqah, furaythah, zifra al-tais, sinam al-tais. Khaisa, sjaharet al-ba’ud, sjaharet al-ghazal, tafar al-tays, khokhawot, mekhisat al-hamr)	[165]	[15,21,22,28]
124		*Ruta chalepensis*	L.	(Eng. Rue, fringed rue)	[166]	[22,24]
125	Salvadoraceae/Salourloruceae	*Salvadora persica*	L.	(Eng. Toothbrush tree, mustard tree, mustard bush) (Arb. Suwak, rak, (‘arak, yeharayk, ehereek)	[167,168]	[15,21,22,24,28]
126	Solanaceae (Nightshade family)	*Withania somnifera*	(L.) Dunal.	(Eng. Ashwagandha, Indian ginseng, poison gooseberry, winter cherry) (Arb. Babu “ebab”, sumal far, haml balbool, morgan, simm, frakh)	[169]	[21]
127	Tamaricaceae (Tamarisk family)	*Tamarix nilotica*	(Ehrenb.) Bunge	(Syn. *Tamarix mannifera* (Ehrenb.) Bunge (h)/Tamarix arabica Bunge) (Eng. Nile tamarisk) (Arb. tarfa’, “terfat”, athl)	[170]	[15,21,22,26,28]
128	Verbenaceae (Verbena, vervain family)	*Clerodendrum inerme*	(L.) Gaertn.	(Eng. Vanajai, garden quinine)	[171]	[15]
129		*Lantana camara*	L.	(Eng. Tickberry)	[172,173]	[15]
130		*Phyla nodiflora*	(L.) Greene	(Syn. *Phyla nodiflora*/Lippia (Phyla) nodiflora (L.) Greene/Phylain nodiflora/Lippia nodiflora (L.) Mychx.) (Arb. Berbin al-jedi, herum dezen, thayyel sini, lebbia, farfakh)	[174]	[15,21]
131		*Vitex agnus-castus* L.	L.	(Eng. Chaste tree)	[175,176]	[20,21,22]
132		*Avicennia marina*	(Forssk.) Vierh.	(Syn. *Avicennia marina* L.) (Eng. Grey mangrove, white mangrove) (Arb. Qurm, gurm)	[177]	[15,21,22,28]
133	Violaceae	*Viola odorata*	L.	(Eng. Sweet violet, garden violet, common blue violet, viol, viotea)	[178,179]	[24]
134	Zingiberaceae (Ginger family)	*Alpinia galanga*	(L.) Sw	(Eng. Greater galangal, thai galangal, blue ginger, thai ginger)	[180,181]	[24]
135		*Zingiber officinale*	Roscoe	(Eng. Ginger)	[182]	[24]
136	Zygophyllaceae (Caltrop, bean-caper, creosote-bush family)	*Peganum harmala*	L.	(Eng. African rue, Syrian rue, wild rue, harmal shrub, harmel, isband, ozallalk, steppenraute)	[183]	[24]
137		*Tribulus parvispinus*	Presl	(Syn. *Tribulus terrestris*) (Eng. Puncture vine, Land caltrops, puncture vine) (Arb. Shershir, kuteb “qatb”, hisek, badl, shuraysah, shiqshiq, dreiss)	[184]	[15,21]

**Table 2:** The UAE EO-bearing species of Asteraceae/Compositae/Anthemideae Family.

No.	Botanical Name (Syn./Eng./Arb. Names)	Form	Life Form	Life Cycle	Economic Value	Folk Medicine	References
1	*Anthemis odontostephana* Boiss. (Arb. O’qhowan)	H	Th	A	Arom, EOs *		[52,186,187]
2	*Artemisia sieberi* Besser (Eng. Wormwood)	H/S	Ch	P	Med, Arom, Eos *, Flav!, Cosm!	(+) (UAE)	[188]
3	*Calendula arvensis* L. (Eng. Field marigold) (Arb. Ain el baqr, eqhwan-asfar, hanwa, hanuwa)	H	Th	A	Med, Fod, Nutr, EOs, Cosm	(+)	[54,189]
4	*Cichorium intybus* L. (Eng. Blue daisy, blue dandelion, blue sailors, blue weed, bunk, coffeeweed) (Arb. hindibaa bareeya, chicoria)	H	Ch	P	Med, Flav, Forg, EOs, Lands	(+) (Europe: “R”: Are aromatic and used with coffee as a substitute) (UAE: “L”: Boiled in water as fever treatment + “Fr”: Eaten to treat headache and boiled in water for treating jaundice)	[55,190]
5	*Conyza bonariensis* (L.) Cronq. (Syn. *Conyza linifolia* (Willd.) Tackh) (Eng. Flax-leaf Fleabane, Wavy-leaf Fleabane, hairy fleabane) (Arb. hashishat el-jebal, tebaq)	H/W	Th	A/B/P	Med, EOs	(+)	[191,192]
6	*Eclipta prostrata* L. (Syn. *Eclipta alba* (L.) Hassk.) (Eng. False daisy, Trailing eclipta ) (Arb. Sa’ada, sauweid, masadate)	H/W	Th	A	Med, Arom, EOs	(+) (China: Herbal medicine) (North Africa: Juice of fresh plant applied to scalp to improve hair growth)	[57]
7	*Grantia aucheri* Boiss.	H	He	A	Med, EOs	(+) (Pakistan: “W”: for snake and scorpion bite)	[58,193]
8	*Launaea nudicaulis* (L.) Hook. f. (Eng. Hawwa Baqrah ara, hindabah ara, huwah ara, naked launea) (Arb. Huwah al ghazal, safara, huwah, hindabah)	H	Ch	A/P	Med, EOs	(+)	[59,60]
9	*Matricaria aurea* (Loefl.) Sch. Bip. (Eng. Golden chamomile) (Arb. Babunaj)	H	Th	A	Med, Arom *, Eos *, Cosm *	(+) (As a carminative and anti-inflammatory) (Used in ointments and lotions) (As a mouthwash against infections of mouth and gums) (chamomile essential oils “true chamomile oil”: for aromatherapy) (UAE: Medicinal tea. “Fl”: To treat abdominal complains)	[194]
10	*Matricaria chamomilla* L. (Syn. *Matricaria recutita*) (Eng. Chamomile, camomile, german chamomile)	H	Th	A	Med, Nutr, Arom, Eos *	(+) (Saudi Arabia: “Fl”: as antibacterial) (Jordan: to treat various diseases “e.g., inflammation and cancer”)	[107,195]
11	*Pluchea arabica* (Boiss.) Qaiser and Lack (Eng. Pluchea) (Arb. godot)	H	Ch	P	Med, Arom, Eos *, Cosm	(+) (UAE: To treat skin and as doedorant) (“W”: Boiled to treat skin ailments + “L”: Extract used as ear drops + “L”: Fresh “L” rubbed on body as deodorant)	[64,66]
12	*Pluchea dioscoridis* (L.) DC. (Syn. *Conyza dioscoridis* (L.) Desf., *Baccharis dioscoridis* L.) (Eng. Ploughmans spikenard, marsh fleabane) (Arb. Sahikee, barnof)	S/T	Ch	P	Med *, Arom, EOs	(+) (Many Important Applications) (UAE)	[65,66]
13	*Pluchea ovalis* (pers.) DC. (Eng. Woolly camphor-weed)	S/T	Ph	A/P	Med *, Arom, EOs	(+) (UAE)	[66]
14	*Pseudognaphalium luteo-album* (L.) H. and B. (Syn. *Gnaphalium luteo-album* L.) (Eng. Cudweed) (Arb. sabount el’afrit)	F/H	Th	A	EOs	(+)	[67]
15	*Pulicaria arabica* (L.) Cass. (Syn. *Inula arabica* L./*Pulicaria elata* Boiss./*Pulicaria laniceps* Bornm.) (Arb. Iqat, abu ‘ain safra)	H	He	A/P	EOs	.	[7]
16	*Pulicaria glutinosa* Jaub. and Spach (Arb. Thal, fal, shajarat fal, muhayda, mithidi, shajarat al-mithidi, shneena, zayyan)	S	Ch	P	Arom *, EOs, Oth *		[7]
17	*Pulicaria inuloides* (Poir.) DC.	S	Ch	P	Med, Fod, Forg, Arom, EOs, Fuel	(+)	[69,70,71]
18	*Pulicaria undulata* (L.) C.A. Meyer (Syn. *Pulicaria crispa* (Forssk.) Benth.) (Eng. Crisp-leaved fleabane) (Arb. Gathgeth, jithjath, ‘urayfijan)	H/S	Ch	A/P	Med *, Fod, Forg, Arom, Eos *, Fuel	(+) (Dropsy, swelling, edema, gout, febrifuges, painkillers) (Egypt: To treat measles and repel insects)	[196]
19	*Rhanterium epapposum* Oliv. (Eng. Rhanterium) (Arb. Arfaj)	S	Ch	p	Forg *, Flav, EOs, Fuel		[197]
20	*Senecio glaucus* L. ssp. coronopifolius (Maire) Al. (Syn. *Senecio desfontanei* Druce) (Eng. Maire alexander, buck’s horn groundsel) (Arb. Qorreis, murair, zamlooq, shakhees, rejel al ghurab)	H	Th	A	Arom, Eos *		[198]
21	*Seriphidium herba-alba* (Asso) Sojak (Syn. Artemisia herba-alba Asso/Artemisia inculta Del) (Eng. Wormwood, white wormwood) (Arb. Ata, ghata, shih)	S	Ph	P	Med, Eos *, FPre!	(+) (Tunisia) (Inhaling smoke thought to be beneficial for both man and animals) (“Sh”: Young “Sh” eaten by mountain travellers) (Many applications) (UAE: “L”: Crushed as a worm treatment and to combat fevers + Many applications)	[76]
22	*Sphagneticola trilobata* (Syn. *Wedelia paludosa* DC.) (Eng. Singapore daisy, creeping-oxeye, trailing daisy, wedelia)	H	Ch	P	Med, EOs, Lands *	(+) (Brazil)	[75,77]

**Table 3:** The UAE EO-bearing species of Fabaceae/Leguminosae/Papilionoideae Family.

No.	Botanical Name (Syn./Eng./Arb. Names)	Form	Life Form	Life Cycle	Economic Value	Folk Medicine	References
1	*Alhagi maurorum* Medik. (Syn. *Alhagi graecorum* Boiss.) (Eng. Camelthorn, camelthorn-bush, caspian manna, persian mannaplant) (Arb. Shwaika, agool, heidj)	H/S	He/Na	P	Med, EOs, Biof	(+) (Infusion of plant or plant juice used to treat worm infestations, cataract, jaundicem migraine, arthritis, constipation) (“R”: Green “R” boiled and taken as tea with lime, as an aphrodisiac and to treat kidney disease) (UAE)	[113]
2	*Lotus halophilus* Boiss. and Spruner (Eng. Greater bird’s foot trefoi) (Arb. Horbeith “hurbuth”, garn al ghazal, ‘asheb al ghanem)	V!/H	Th	A/P	Med, Fod, Forg, EOs, Lands	(+) (Qatar: as tonic and sedative)	[79,89]
3	*Medicago polymorpha* L. (Eng. California burclover, toothed bur clover, toothed medick, burr medic)	F/W	Th	A	Med *, Fod, Forg, EOs, Eco, Lands	(+) (India: for medicinal purposes for skin plagues and dysentery) (Bolivia: for medicinal purposes since 16 century) (Italy: for treating rheumatic pains, wounds and still used until today)	[115]
4	*Medicago sativa* L. (Eng. Alfalfa, lucerne)	H	He	P	Med *, Forg *, EOs, Eco	(+) (Great therapeutic benefits) (Used for boosting energy levels)	[116]
5	*Rhynchosia minima* (L.) DC. (Eng. least snout-bean, burn-mouth-vine and jumby bean) (Arb. Baql)	V/H	Ch	P	Med, EOs	(+) (Saudi Arabia: Used as abortive) (UAE)	[116,117]
6	*Tephrosia persica* Boiss. (Syn. *Tephrosia apollinea* (Delile) DC.) (Arb. Dhafra, omayye, nafal)	H/S	Ch	P	Med, Arom, Eos *	(+) (“L”: Boiled with water used as eardrops for earache + “Bk”: Powdered and mixed with water put into camel’s ears to remove ticks + “L”: Powdered, heated and mixed as a paste with water and/or salt and applied on wounds and fractures to relieve pain) (UAE)	[118]
7	*Trigonella hamosa* L. (Eng. Branched fenugreek, egyptian fenugreek) (Arb. Nafal, qutifa, qirqas, darjal, eshb al-malik, qurt)	H	Th	A	Med, Forg, Flav, EOs	(+)	[79]
8	*Ononis sicula* Guss.	H	Th	A	EOs	.	[119]
9	*Acacia nilotica* (L.) Delile (Syn. *Acacia Arabica* (Lam.) Willd.) (Eng. Gum arabic tree, babul/kikar, egyptian thorn, sant tree, al-sant, prickly acacia) (Arb. Sunt garath “kurut”, babul, tulh. Fruit: karat)	T	Ph	P	Med *, EOs, Lands	(+) (Pearl drivers used to apply an infusion of fruits to skin after dives) (“L”: Poultice of “L” used to treat joint pains) (Resin mixed with egg-white applied to eyes to treat cararacts) (“L”: Eaten to treat diarrhoea) (“Se”: Soaked in water or milk drunk to treat diabetes) (“Pd”: Smoke from burning “Pds” inhaled for colds) (UAE: Applied to soothe burns. “L”: are pounded into a paste and used a poultice on boils and swellings or applied around boils to draw out the pus)	[120]
10	*Acacia tortilis* (Forssk.) Hayne (Eng. Umbrella thorn) (Arb. Samr “samur”, salam)	S/T	Ph	P	Med, Forg, EOs	(+) (Mostly yields resin, used as a gum to heal wounds)	[122]
11	*Prosopis farcta* (Banks and Sol.) Mac. (Eng. Dwarf mesquite, syrian mesquite) (Arb. Yanbut, agoul, awsaj)	S	Ch	P	EOs	.	[123,128]

**Table 4:** The UAE EO-bearing species of Lamiaceae/Labiatae.

No.	Botanical Name (Syn./Eng./Arb. Names)	Form	Life Form	Life Cycle	Economic Value	Folk Medicine	References
1	*Lallemantia royleana* (Benth.) Benth. (Eng. Bian bing cao)	H	Th	A	Med *, Eos *	(+)	[128]
2	*Mentha spicata* (Eng. Spearmint, spear mint)	H	He	P	Med, Fod, Flav, Arom *, Eos *	(+) (UAE)	[199]
3	*Ocimum forsskaolii* Benth. (Syn. *Ocimum forskolei* Benth.) (Eng. Rehan, sawma) (Arb. Basil)	H	Th	A/P	Med *, Arom, EOs, Insec, Lands, Oth	(+) (Oman: “L”: as deodorant + “L”: Fragnance eases headaches and dizziness + “L”: Crushed and placed in nose to treat colds and in ears to treat earaches + “L”: Juice from young “L” as eye drops or to soothe insect bites) (UAE: “L”: to treat vomiting, against itching)	[69,131]
4	*Salvia aegyptiaca* L. (Eng. Egyptian sage) (Arb. Ra’al, na’aim, ghbeisha, shajarat al ghazal, khizam)	H	Ch/Th	A/P	Med *, Eos *	(+) (To treat diarrhoea, gonorrhoea and haemorrhoids) (As demulcent, antispasmodic, cicatrizant, antiseptic and stomachic) (Its non-polar extracts have been tested as antimicrobial) (Nutlets are used in a drink to treat diarrhoea and piles) (UAE)	[2]
5	*Salvia macilenta* Boiss. (Eng. Khizama) (Arb. Khmayzah lethnay, bithman)	H	Ch	P	Med, Eos *	(+)	[3]
6	*Salvia macrosiphon* Boiss. (Arb. Shajarat Al Ghazal)	H	He	P	Med, Eos *	(+)	[140,141]
7	*Salvia mirzayanii* Rech.f. and Esfandiari	H	Ch	P	EOs	.	[200]
8	*Salvia spinosa* L. (Arb. Shajarat al-ghazal)	H	He	P	Eos *	.	[136]
9	*Teucrium polium* L. (Eng. Felty germander)	H/S	Th	P	Med *, Arom, EOs	(+) (to treat liver diseases, antispasmodic, antidiabetic and lowering blood lipid) (Many medicinal uses: to treat malaria, insect bites and abscesses) (UAE: “L” and “St”)	[137]
10	*Teucrium stocksianum* Boiss. (Eng. Jadah, yadah, Ja‘adah) (Arb. Ya’dah, brair)	H	Ch	P	Med *, Eos *	(+) (Many applications in medicine) (Antispasmodic activity) (UAE: Many medicinal applications. Antispasmodic activity. To treat kidney, stomach pains, thyroids problems, common cold)	[6]
11	*Zataria multiflora* Boiss. (Eng. Za’atar, shirazi thyme)	H	Ch	P	Med, Flav, Arom, Eos *	(+) (UAE: to treat cold, indigestion, toothache)	[141]

**Table 5:** Location of the UAE EO-bearing plants of Asteraceae/Compositae/Anthemideae.

No.	Botanical Name	Emirates	Important Locations	Soil	Habitats	Flowering	Wildlife Status (Past) vs. (Present)	References
1	*Anthemis odontostephana* Boiss.	(RAK, F)	(RA)	(Sil, Roc)	(Mou)	Feb. to Apr.	(NC) (CO)	[15,21]
2	*Artemisia sieberi* Besser	.	.	.	.	.	(CO)	[28]
3	*Calendula arvensis* L.	(RAK, F, S)	(HM, RA)	(Sil, Roc)	(FF, Mou)	Jan. to Mar. June to Nov.	(CO) (NC, CO)	[15,21]
4	*Cichorium intybus* L.	(RAK)	.	(San)	(FF)	Feb. to Apr.	(CO, RA) (RA)	[15,21,28]
5	*Conyza bonariensis* (L.) Cronq.	(AD)	(SL)	.	(Oas, Gar, Plat)	Jan. to May.	(CO)	[21,22]
6	*Eclipta prostrata* L.	(F)	(SL)	.	(Plat)	Dec. to Apr.	(NC) (NC)	[15,21]
7	*Grantia aucheri* Boiss.	(AD)	(Ain)	.	(Rod, Wad)	Jan. to Apr.	.	[27]
8	*Launaea nudicaulis* (L.) Hook. f.	(AD, Du)	(SL)	(San)	(Oas, Plat)	Feb. to Apr.	(CO) (NC)	[15,21,22]
9	*Matricaria aurea* (Loefl.) Sch. Bip.	(RAK, F)	(RA)	(Sil, Roc)	(PX, Mou)	Feb. to Apr.	(CO) (CO)	[21,28]
10	*Matricaria chamomilla* L.	.	.	.	(Rod!, AF!)	.	.	[63]
11	*Pluchea arabica* (Boiss.) Qaiser and Lack	(RAK)	.	(Roc)	(Wad, Plat, Wat)	Feb. to Apr.	(NE) (RA)	[28]
12	*Pluchea dioscoridis* (L.) DC.	.	(WC)	(San, Sal)	(Cos, Oas, Gar, Urb, AF)	Through the year.	(CO) (CO)	[15,21,22]
13	*Pluchea ovalis* (pers.) DC.	(Du)	.	.	.	.	(NC)	[15,21]
14	*Pseudognaphalium luteo-album* (L.) H. and B.	(AD)	(Ain)	.	(Plat)	Feb. to May.	(CO)	[21,22]
15	*Pulicaria arabica* (L.) Cass.	(S, F, RAK)	(HM)	.	(Wad, Wat, Plat)	Feb. to Apr.	(NC, CO) (NC, CO)	[21]
16	*Pulicaria glutinosa* Jaub. and Spach	(F, RAK, S, AD)	(HM)	(Roc)	(Plat, Mou)	Feb. to Jun.	(CO) (CO)	[15,21,22]
17	*Pulicaria inuloides* (Poir.) DC.	.	.	.	.	.	.	[21]
18	*Pulicaria undulata* (L.) C.A. Meyer	(RAK, AD)	(SL)	(San, Sil)	(Apl, AF)	Apr. to Jul. Mar. to Aug.! Mar. to June!	(FC) (FC)	[15,21,22]
19	*Rhanterium epapposum* Oliv.	(AD)	(Ain, CC, EE, NE)	(San, Roc)	(Dun, Plat, Apl, Hil)	Jan. to May.	(CO)	[15,21,22]
20	*Senecio glaucus* L. ssp. coronopifolius (Maire) Al.	(RAK, UAQ, AD)	(CN)	(San, Sal)	(Cos, FF)	Feb. to Apr.	(CO) (CO)	[15,21,22]
21	*Seriphidium herba-alba* (Asso) Sojak	(RAK, F)	(RA)	(Roc)	(PX, Mou)	Feb. to Apr.	(NC) (CO)	[21]
22	*Sphagneticola trilobata*	.	.	.	.	Through the year. Spring to Autumn!	.	[15]

**Table 6:** Location of the UAE EO-bearing plants of Fabaceae/Leguminosae/Papilionoideae.

No.	Botanical Name	Emirates	Important Locations	Soil	Habitats	Flowering	Wildlife Status (Past) vs. (Present)	References
1	*Alhagi maurorum* Medik.	(AD)	(SL)	(Sal!)	(Cos, Dun, Rod, DS)!	Mar. to Aug. Apr. to Jul!	(CO) (CO)	[15,24]
2	*Lotus halophilus* Boiss. and Spruner	(AD, RAK)	(WC)	(San, Sal)	(Cos, Dun)	.	(NC) (CO)	[15,21,22]
3	*Medicago polymorpha* L.	(Du, AD)	(SL)	(Roc)!	(Gar, Plat, Mou, DS)!	Feb. to Apr.!	(CO)!	[15,21,22]
4	*Medicago sativa* L.	(Du)	.	.	.	.	(NC)	[15]
5	*Rhynchosia minima* (L.) DC.	(AD)	(NE)	(San, Roc)	(Dun, Wad, Mou)	Feb. to May.	(LC)	[15,21,22]
6	*Tephrosia persica* Boiss.	(F, S, RAK)	(RA, HM)	(San, Roc)	(Wad, Mou “low”)	Jan. to May.	(NC) (CO)	[15,21]
7	*Trigonella hamosa* L.	(Du, AD)	(SL)	(San)	(Dun, Oas, Gar, Plat)	Feb. to Apr.	(FC) (NC)	[15,21,22]
8	*Ononis sicula* Guss.	.	.	.	.	.	.	[21]
9	*Acacia nilotica* (L.) Delile	(AD)	(EE, NE)	(Roc)	(Oas, Wad, Plat, Gar, Mou)	Mar. to Nov. Nov. to Apr.!	(CO) (CO)	[15,21,22,28]
10	*Acacia tortilis* (Forssk.) Hayne	(AD, S)	(EE)	(San, Roc)	(Pla, Dun. Wad, Mou “medium elevations”)	Apr. to Jun. Mar. to July!	(FC) (CO)	[15,21,22]
11	*Prosopis farcta* (Banks and Sol.) Mac.	(AD)	(Ain, SL)	(San, Roc)	(Dun, Plat, Mou)	Apr to Aug. Apr to July!	(NC) (RA)	[15,21,22]

**Table 7:** Location of the UAE EO-bearing plants of Lamiaceae/Labiatae.

No.	Botanical Name	Emirates	Important Locations	Soil	Habitats	Flowering	Wildlife Status (Past) vs. (Present)	References
1	*Lallemantia royleana* (Benth.) Benth.	(RAK, F)	(RA)	(Roc)	(Mou “medium and high elevations”)	Feb. to Apr.	(RA)	[21]
2	*Mentha spicata*	.	.	.	.	.	.	[63,130]
3	*Ocimum forsskaolii* Benth.	(S, F, RAK)	(HM, RA)	.	(Plat)	Feb. to Apr.	(NE) (NC)	[15,21,28]
4	*Salvia aegyptiaca* L.	(AD, S, F, RAK)	(HM)	(Roc)	(Wad, Hil “all elevations”, Mou)	Feb. to May.	(CO) (CO)	[15,21,22,24,28]
5	*Salvia macilenta* Boiss.	(F, RAK, AD)	(EE)	(Roc)	(Plat, Wad, Hil “low”, Mou)	Feb. to May.	(CO) (NC, CO)	[15,21,22]
6	*Salvia macrosiphon* Boiss.	(F, S !, RAK!)	(RA! HM!)	(Roc)	(Wad, Hil, Mou)	Feb. to May.	(RA)	[15,21]
7	*Salvia mirzayanii* Rech.f. and Esfandiari	(F, RAK)	(RA)	(Roc)	(Wad, Hil “low”, Mou)	Feb to May!	(NC)!	[21]
8	*Salvia spinosa* L.	(AD, S, F, RAK)	(HM, RA)	(Roc)	(Rod, Wad, Hil “low to medium elevations”, Mou)	Feb. to Apr.!	(NC, CO)	[21,22]
9	*Teucrium polium* L.	(F, RAK)	(RA)	.	(Wad, Hil “all elevations”)!	Feb. to May.!	(NE) (RA)!	[21,28]
10	*Teucrium stocksianum* Boiss.	(F, S, RAK, AD)	(HM, RA, KF)	(Roc)	(Wad, Hil “all elevations”, Mou)	Mar. to Apr.! Feb. to May.	(CO) (NC, CO)	[15,21,22,24,28]
11	*Zataria multiflora* Boiss.	.	.	.	.	.	.	[24]

**Table 8:** Details of EOs isolated from species of Asteraceae/Compositae/Anthemideae.

No.	Botanical Name	Plant Part	Physical Properties	Yield (%)	Isolation Method	Main Chemical Groups/Components	Biological Activity	References
1	*Anthemis odontostephana* Boiss.	Fl/L/St/R	(Yellow color/Aromatic odor)Fl,HD	(0.2) Fl,HD (0.7) Fl,HD (0.5)L,HD (0.7) St,HD (0.2) R,HD	HD	(Monoterpene hydrocarbons, oxygenated monoterpenes, sesquiterpene hydrocarbons, oxygenated sesquiterpenes, phenylpropanoids)Fl,HD (Spathulenol, hexadecanoic acid, germacrene D, 1,8-cineole, 6-methyl-5-hepten-2-one, caryophyllene oxide, β-caryophyllene, camphor) Fl,HD (Borneol) Fl/L/St,HD (Pentadecanoic acid)R,HD	[AB/AF/AM]Fl/L/St/R,HD (AB: Gram-negative bacteria: *Escherichia coli*, *Escherichia coli*, *Klebsiella* bacteria. Gram-positive bacteria: *Staphylococcus aureus*, *Staphylococcus epidermidis*, *Corynebacterium glutamicum*)Fl/L/St/R,HD (AF: *Aspergillus niger*, *Fusarium solani* species complex, *Alternaria alternata*)Fl/L/St/R,HD	[52,186,187]
2	*Artemisia sieberi* Besser	L/Sh	Fresh herbaceous, camphoraceous, earthy odor with a fruity and dried plum-like background	(1.7)Sh,HD (1.02)Sh,SD (0.5 to 3.5)Sh,HD (1.6 to 14.0)Sh,SFE	HD/SD/SFE	(Sesquiterpenes: dehydro-1,8-sesquicineole)Sh (camphor, camphene, 1,8-cineol, β-thujone, α-pinene)Sh,HD (camphor, 1,8-Cineol)Sh,SFE (α- thujone, β- thujone, camphor) (camphor, 1,8-cineole, bornyl acetate, neryl acetate)Sh,SD (camphene, 1, 8-cineole, trans-thujone, camphor, borneol)Sh,HD (camphor, 1,8-cineole, bornyl acetate)Sh,SD (camphor, camphene, 1,8-cineol, β-thujone, α-pinene)L,HD (camphor, 1,8-cineole, camphene, terpinen-4-ol, α-terpineol, dehydro-1,8-sesquicineole)Sh (ketone, 1, 8 cineole, selin-11-en-4-a-ol, lavandulon)Sh (1,8cineol, myrcene, 1,8cineol, Eudesm-7(11)-en-4-ol, 4-tepinyl acetate, davanone, p-cymene)	[AB/AF/AM/FT/AD] (AB: Gram-positive bacteria, Gram-*negative bacteria*) (AM: yeast and fungi) (AM: Gram-positive bacilli: *Listeria monocytogenes*, *Bacillus cereus*. Gram-positive cocci: *Streptococcus mutans*) (AB: *Pseudomonas aeroginosa*, *Staphylococcus aureus*, *Escherichia coli*)Sh (FT against insects: *Callosobruchus maculatus, Sitophilus oryzae, Tribolium castaneum*) L,HD (AF: for patient with *Pityriasis versicolor*)	[188]
3	*Calendula arvensis* L.	Sh	.	(0.02 to 0.06)Sh,HD	HD	(γ-cadinene, α-cadinol)Sh,HD	.	[54,189]
4	*Cichorium intybus* L.	Sh/Fl	(Yellow color/Strong odor)Sh, HD	.	HD	(carvacrol, thymol, cinnamic aldehyde, camphor, carvone, linalool, α-terpineol)Sh,HD	.	[55,190]
5	*Conyza bonariensis* (L.) Cronq.	W/Sh/Fl	.	(0.22)W, SD	HD/SD	(Sesquiterpenes)HD (Monoterpenes, acetylenes, sesquiterpenes, diterpenes)W,SD (matricaria methyl ester, limonene, manool, carvone)W,SD ((E)-β-farnesene, germacrene D, β-caryophyllene, limonene)HD (matricaria ester, (Z)-nerolidol, caryophyllene oxide)Sh (matricaria ester, caryophyllene oxide, (E)-β-farnesene)Sh (matricaria ester, geranyl acetone, trans-α-bergamotene, limonene)Sh	[AB/AF/AM/IS]HD	[191,192]
6	*Eclipta prostrata* L.	L/St/Fl/Sh	(Yellow color)Sh, HD	(0.1)Sh,HD	HD	(Sesquiteprenoids, straight chain hydrocarbons, monoterpenoids, P-caryophyllene, a-humulene) (hydrocarbons with sesquiterpene predominating, alcohols, ketones, aldehydes, oxides, esters)Sh,HD (α-Humulene, 6,9-heptadecadiene, (E)-β-farnesene, α-phellandrene)Sh,HD (sesquiterpenoids)L (sesquiteprenoids, straight chain, hydrocarbons, monoterpenoids)St (P-caryophyllene)L (a-humulene, (E)-beta-farnesene)St	.	[57]
7	*Grantia aucheri* Boiss.	Sh	.	(0.53)Sh,HD	HD	(Sesquiterpenes: himachalol)Sh,HD	.	[58,193]
8	*Launaea nudicaulis* (L.) Hook. f.	Sh	.	.	SD	(limonene, Z-citral, E-citral)Sh,SD	[AB/AM] (AB: Gram-positive bacteria: *Staphylococcus aureus*. Gram-negative bacteria: *Escherichia coli*)Sh,SD	[59,60]
9	*Matricaria aurea* (Loefl.) Sch. Bip.	Fl	.	(0.63)Fl,HD	HD	(α-bisabolene oxide A, α-bisabolol oxide A, chamazolene)Fl,HD	.	[194]
10	*Matricaria chamomilla* L.	Sh/Fl	(Dark blue color/Strong characteristic odor)Fl,HD	(0.626 to 0.754)Fl,HD (0.25)Fl,SD (0.73)Fl,HD (4.33)Fl,SFE	HD/SD/SDE/SFE	(azulene-7-ethyl-1,4-dimethyl, limonene, bisabolol oxides A and B, bisabolone oxide, trans-β-farnesen, isobornyl isobutyrate<8-isobutyryloxy>)Fl,HD (α-bisabolol, trans-trans-farnesol, cis-β-farnesene, guaiazulene, α-cubebene, α-bisabolol oxide A, chamazulene)Fl,SD (Guaiazulene, (E)-β-faranesens, chamazulene, α-bisabolol oxide B, α-bisabolol, hexadecanole)Fl,HD (Trans-anethole, estragole, fenchone, limonene)Fl,HD ((-)-α-bisabolol, chamazulene, (-)-α-bisabololoxides)Fl (chamazulene, cis-spiroether, trans-spiroether)Fl,SD (α-bisabolol oxide A and B, (E)-β-farnesene, α-bisabolol, chamazulene)Fl,HD (Sesquiterpenoid)Fl,SDE (Bisabolol oxide, bisabolon oxide, β-farnesense, α-bisabolol, chamazulene and en-yn-dicycloether)Fl,SDE (bisabolol oxide A, α-bisabolol, bisabolol oxide B, cis-enyne-bicycloether, bisabolon oxide A, chamazulene, spathulenol, (E)-β-farnesene) (β-farnesene, α-farnesene, γ-cadinene, α-bisabolol oxide B, α-bisabolol, chamazulene, α-bisabolol oxide A, cis, trans-dicycloether)Fl,SFE ((E)-β-farnesene, guaiazulene, α-bisabolol oxide A, α-farnesene, α-bisabolol)Sh	[AB/AF/AM/AO/AS] (AF: *Aspergillus niger*)Fl,SD (AB: *Streptoccus pygenes*, *Streptococcus mutans*, *Streptococcus salivarius, Streptococcus faecalis*, *Streptococcus sanguis*)Fl,HD (AM: *Aspergillus flavus*, *Candida albicans*, *Bacillus cereus*, *Staphylococcus aureus*)Fl,HD	[107,195]
11	*Pluchea arabica* (Boiss.) Qaiser and Lack	Sh/Fl	.	(0.08)Sh,SD	SD	(Sesquiterpene)Sh,SD (δ-cadinol, 9-(1-methylethylidene)-bicyclo[6.1.0]nonane, caryophyllene oxide, methyleugenol, β-caryophyllene)Sh,SD (godotol A and godotol B)	[AB/AM] (AB: *Staphylococcus aureus*, *Candida albicans*, *Bacillus subtilis)*Sh,SD	[64,66]
12	*Pluchea dioscoridis* (L.) DC.	L	.	.	HD	(Monoterpenes, light oxygenated compounds, sesquiterpenes, heavy oxygenated compounds)L,HD (Farnesol cis-trans, farnesol, nuciferol, trans-cadinol, eudesmol, methyl eicosane)L,HD (Farnesol, uiterpene alcohols, oxygenated sesquiterpenes, sesquiterpene hydrocarbons)	[AO/AB/AM]!L!,HD! (AB: Gram-positive bacteria; Gram-negative bacteria)!L!,HD! (AM: *Mycotic infection* with *C. albicans*)!L!,HD!	[65,66]
13	*Pluchea ovalis* (pers.) DC.	L	.	(0.02)L,SD	SD	(limonene, p-cymene, ß-maaliene, ß-phellandrene, isocomene Laggera aurita, 2,5-dimetoxy-p-cymene, ß-caryophyllene, δ-cadinene, α-cadinol)L,SD	.	[66]
14	*Pseudognaphalium luteo-album* (L.) H. and B.	Sh!	.	.	MD	(Monoterpene hydrocarbons, oxygenated monoterpenes, sesquiterpene hydrocarbons, oxygenated sesquiterpenes, liphatic compounds, fatty acids, esters)Sh!,Microdistillation! (decanal, β-caryophyllene, α-gurjunene)Sh!,Microdistillation!	.	[67]
15	*Pulicaria arabica* (L.) Cass.	Sh	.	.	SD	(Sesquiterpene hydrocarbons, alcohols)Sh,SD		[7]
16	*Pulicaria glutinosa* Jaub. and Spach	Sh/Fl	.	(0.5)Sh,SD	SD	(sesquiterpenes)Sh,SD (p-elemene, 7-cadinol, a-cadinol)Sh,SD		[7,197]
17	*Pulicaria inuloides* (Poir.) DC.	L/Sh/W	(Strong odor)W, HD	(0.5)W,HD	HD/SD	(2-Cyclohexen-1-one, 2-methyl-5-(1-methyl), Benzene, methyl-, Z.citol)L,HD (2-Cyclohexen-1-one, 2-methyl-5-(1-methyl) with Hexadecanoic acid (CAS), Ethane, 1,2-diethoxy)W,HD (2-cyclohexen-1-one, 2-methyl-5-(1-methyl), benzene, methyl-)Sh,SD	[AB/AM]Sh,HD!/SD! (AB: G+: *Staphylococcus aureus*, *Streptococcus pneumoniae*, *Bacillus subtilis*; G-: *Escherichia coli*)Sh,HD!/SD! (AM: against yeast: *Candida albicans*)Sh,HD!/SD!	[69,70,71]
18	*Pulicaria undulata* (L.) C.A. Meyer	Sh	.	(2.5)Sh,SD (0.32)Sh,HD	HD/SD	(Phenolic compounds, monoterpene hydrocarbons, low in sesquiterpene hydrocarbons)Sh,SD (oxygenated monoterpenes:carvotanacetone. Sesquiterpene lactone, δ-cadinene, α-elemene, sabinol) (1,8-cineole)Sh,HD ((+)-carvotanacetone)Sh,SD	AB!/R!	[196]
19	*Rhanterium epapposum* Oliv.	Sh!/Fl!	.	0.25!	HD!	(Terpenoids, Non-terpenoid aliphatic and aromatic structures) (terpenoids: α-phellandrene, linalol, geraniol, bulnesol) (α-phellandrene, linalol, geraniol, bulnesol, β-phellandrene)	.	[197]
20	*Senecio glaucus* L. ssp. coronopifolius (Maire) Al.	Sh!/Fl!/Fr!	(Apricot-like odor “while the odor of the intact plant is herbaceous, spicy and floral fruity”)SD/H	.	SD/H	(Monoterpenes,Sesquiterpenes)SD/H (myrcene, dehydrofukinone)SD/H	.	[198]
21	*Seriphidium herba-alba* (Asso) Sojak	L/Fl/Sh	(Yellow color)L/Fl,HD	(1.45)L/Fl, HD	HD	(Oxygenated monoterpenes, oxygenated sesquiterpenes)L/Fl,HD (cis-chrysantenyl acetate, the sabinyl acetate and the α-thujone)L/Fl,HD	[AB/AF/AM]Sh,HD [AO/AM]L/Fl,HD (AM: *S. typhimurium*, *E. coli*, *K. pneumoniae*, *P. aeruginosa*, *E. faecalis*, *B. cereus*, *F. solani*, *A. oxysporum*)L/Fl,HD	[76]
22	*Sphagneticola trilobata*	L/St/Fl/Sh	.	(0.48 to 0.78)Sh, HD (0.18 to 0.25)Sh, HD (0.09)	HD/SD	(Hydrocarbon sesquiterpenes, hydrocarbon monoterpenes, low levels of oxygenated sesquiterpenes)Sh,HD (α-pinene, β-pinene, limonene, γ-muurolene)L (germacrene D, α-phellandrene, α-pinene, E-caryophyllene, bicyclogermacrene, limonene, α-humulene)Sh,HD (α-pinene, α-phellandrene, sabinene, limonene, β-pinene, camphene, 10-nor-calamenen-10-one, germacrene D, γ-amorphene)Sh,HD	.	[75,77]

**Table 9:** Details of EOs isolated from species of Fabaceae/Leguminosae/Papilionoideae.

No.	Botanical Name	Plant Part	Physical Properties	Yield (%)	Isolation Method	Main Chemical Groups/Components	Biological Activity	References
1	*Alhagi maurorum* Medik.	L/St	.	.	DSD	(Ketones, acid derivatives, terpenoids, hydrocarbons)L/St,DSD (heterocyclics compunds)L,DSD (drimenol, 9-octylleptadecane, 4-hexyl-2,5-dioxo-3-furanacetic acid, 2-nonadecanone, pentacosane)L,DSD (Aldehydes)St,DSD (neophytadiene,trans-b-ionone, 6,10,14-trimethyl-2-pentadecanone, actinidiolide, nonacosane)St,DSD (drimenol, octadecane, eicosane, docosane, tetracosane, squalene)L/St,DSD	.	[113]
2	*Lotus halophilus* Boiss. and Spruner	Sh	(Yellow color)Sh,SD	(0.07 “fresh weight basis”)Sh,SD	SD	(phytol, Heptadecane, 2,9-Dimethyldecane)Sh,SD	.	[79,89]
3	*Medicago polymorpha* L.	.	.	(0.5)HD	HD	(Terpenoids, alcohols, ketones, aldehydes, esters, hydrocarbons, high amount of fatty acids, benzene compounds)HD (Undecanoic acid, 2-dodecanone, hexadecanoic acid, oleic acid, tetracosane)HD	.	[115]
4	*Medicago sativa* L.	Fl	.	.	TT	(Alcohols, esters, ketones, terpenes, furanoids)Fl,TT (Trans-2-hexenal)Fl,TT	.	[116]
5	*Rhynchosia minima* (L.) DC.	L	.	(0.18)L,SD	SD	(isopropyl toluene, O-cymene, camphene, limonene, 2- β-pinene, α-terpinolene, α-pinene, myrcene)L,SD	[AB] [AB/AF/AM/AO]L,SD (AB: *A. calcoaceticus*, *B. subtilis*, *C. freundii*, *C. sporogenes*, *Escherichia coli*, *P. vulgaris*, *P. aeruginosa*, *S. typhii*, *Staphylococcus aureus*, *Y. enterocolitica*)L,SD (AF: *Candida albicans*, *A. niger*, *A. flavus*, *P. notatum*)L,SD	[116,117]
6	*Tephrosia persica* Boiss.	L /St	.	(0.05 “fresh weight basis”)L/St,HD	HD	(Sesquiterpenoids, monoterpenoids)L/St,HD (germacrene D, spathulenol, caryophyllene oxide, trans-β-caryophyllene)L,HD (germacrene D, geyrene, trans-β-caryophyllene, spathulenol, caryophyllene oxide)St,HD	(AM/IS)!	[118]
7	*Trigonella hamosa* L.	Sh	(Yellow color)Sh,SD	(0.04 “on fresh weight basis”)Sh,SD	SD or HD!	(Palmitic acid, tetradecanoic acid, linolenic acid methyl ester, phytol, decanoic acid)Sh,SD	.	[79]
8	*Ononis sicula* Guss.	.	.	.	.	(Oxygenated sesquiterpenes, sesquiterpene hydrocarbons) (sesquiterpene hydrocarbons: selin-11-en-4-α-ol, α-selinene)	[AO]	[119]
9	*Acacia nilotica* (L.) Delile	St/Pd/Bk	.	(0.08)St,HD (4.56)Pd,SFE(4.86,5.05)Bk,SFE	HD/SFE	(Monoterpenoid compounds, sesquiterpenes)St,HD (Monoterpenoid compounds: menthol, limonene. α-Curcumene, carvacrol)St,HD	[AB/AF/AM] (AB: *Bacillus subtilis)*Pd!/Bk!,SFE (AF: *Ganoderma lucidum*)Pd!/Bk!,SFE	[120]
10	*Acacia tortilis* (Forssk.) Hayne	L	(Yellow–green color)L,GC-FID	(0.12)L,GC-FID	GC-FID	(Monoterpenes, rich sesquiterpenoid compounds)L,GC-FID (α-humulene, α-cadinol, nerolidol, γ-cadinene, 2-(E)-octenal)L,GC-FID	.	[122]
11	*Prosopis farcta* (Banks and Sol.) Mac.	St/Pd/L/Fl/Se/R/W	(Pleasant odor)W,SD	(0.00472 to 0.00793)W, SD	SD	(Saturated hydrocarbons, unsaturated hydrocarbons, aldehydes, carboxylic acids)Sh,SD (Heneicosane, 6,10,14-Trimethylpentadecan-2-one, Docosane, 2-Methyl-1-tertiobutilprop-1,3-yl-, D-Limonene, Methyl hexadecanoate)St,SD (Phytol, Benzyl benzoate, 3-Hydroxy-beta-damascone)L,SD (Phytol, Tetradeca-1,13-diene, Eicosane)Fl,SD (Methyl octadec-9-enoate, Phytol, Methyl hexadecanoate)Pd,SD (Octadecanal, Hexadecanal, Heptadeca-1, 11,13-triene)R,SD	.	[122,128]

**Table 10:** Details of EOs isolated from species of Lamiaceae/Labiatae.

No.	Botanical Name	Plant Part	Physical Properties	Yield (%)	Isolation Method	Main Chemical Groups/Components	Biological Activity	References
1	*Lallemantia royleana* (Benth.) Benth.	L/St/Fl/Sh	.	.	HD	(trans-pinocarvyl acetate, pinocarvone, β-pinene, (E)-β-ocimene, terpinolene, linalool, trans- pinocarveol, 3-thujen-2-one, myrtenal, verbenone, trans-carveol, cis-carveol, pulegone, carvacrol, dihydrocarvyl acetate, β-cubebene)Sh,HD	[ AB/AF/AM]Sh,HD (AB: *Staphylococcus aureus*, *Bacillus subtilis*, *Klebsiella pneumoniae*)Sh,HD (AF: *Candida albicans*, *Aspergillus niger*)Sh,HD	[128]
2	*Mentha spicata*	L/Sh	(Light green color)L,HD	(0.53)Sh,HD (0.566 ± 0.02 “on fresh weight basis”)L,HD (1.2)Sh,HD!orSD! (0.1 to 1.8)Sh!orL! (0.9)Sh,SD (0.32)Sh,SD SFE	HD/SD/SFE	(Oxygenated, non-oxygenated monoterpenes, sesquiterpenes)SD (carvacrol, thymol) (Carvone, Trans carveol)Sh,HD (piperitone oxide, piperitenone oxide, carvone, dihydrocarvone)L,HD (carvone, limonene, 1,8-cineole, trans-carveol)L,HD (carvone, cis-carveol, limonene, 1,8 cineol, cis-dihydrocarvone, carvyl acetate, cis-sabinene hydrate)Sh,HD!or SD! (carvone, menthone)HD (linalool, germacrene D, β-caryophyllene, 1,8 cineole)Sh!orL! (menthol, menthone) (carvone, limonene, 1,8-cineole, menthone, linalool, isomenthone)Sh,SD (piperitenone oxide)Sh,SD (menthol, carvone, D-Limonene)L,HD (piperitenone oxide)Sh,HD (carvone, cis-carveol, limonene)L (Carvone, Limonene, Cineole, Linalool, Menthol, Dihydrocarvone)SD (Carvone, Limonene, a-pinene, Cineole, Linalool, Menthol, Dihydrocarvone)SFE	[AB/AF/AM/AO/IS/MP]Sh,HD!orSD! [IS/LA/MR]L (LA and MR: against *Culex quinquefasciatus*, *Aedes aegypti*, *Anopheles stephensi*)L (AO: good activity)Sh,HD!orSD! (AO: good activity)L,HD (strong AB: *Staphylococcus aureus*, *Escherichia coli*, *Bacillus subtilis*, *Pasturella multocida*)Sh,HD!orSD! (strong AF: *Aspergillus niger*, *Mucor mucedo*, *Fusarium solani*, *Botryodiplodia theobromae*, *Rhizopus solani*)Sh,HD!orSD! (AM: *Enterococcus faecium*, *Salmonella cholerasuis*, *B. subtilis*)Sh,SD	[199]
3	*Ocimum forsskaolii* Benth.	L/Fl/Sh	.	(0.45 to 0.47)L,SD (0.6 to 0.96)Fl,SD	HD/SD/H	(estragole, linalool)L/Fl/Sh,SD (linalool, methyl chavicol, (E)-methyl cinnamate, myrcene, eugenol) ((R)-(-)-linalool, (S)-(+)-1-octen-3-ol, trans-caryophyllene, naphthalene, methyl salicylate, (R)-(-)-a-copaene, methyl cinnamate, (E)-ocimene) (benzene, methyl-)Sh,SD (Bicyclo hept-2-ene, 2, naphthalene, phytol)Sh,SD	[AB/AF/AM]L,SD [AO]Sh,SD (MR: against female *Anopheles gambiae*)Sh,HD (MR: *Aedes aegypti*)H (weak AF: against Dermatophytes)L,SD (AM: *Candida albicans*)Sh,SD	[69,131]
4	*Salvia aegyptiaca* L.	W	(Yellow color/same plant odor)W,SD	(0.033!)W, SD	SD	(Terpenoidal constituents, fat derivatives)W,SD (Aristolene, diphenyl amine, methyl palmitate)W,SD	.	[2]
5	*Salvia macilenta* Boiss.	Sh/W	.	.	HD	(Rich in monoterpene hydrocarbons)Sh,HD (γ-elemene, thymol, elemol, β-caryophyllene)Sh,HD	.	[3]
6	*Salvia macrosiphon* Boiss.	Sh	(Yellow color)Sh,HD	(0.14)Sh, HD (0.14 to 0.23)Sh, HD (0.5)Sh,HD	HD/SD	(Sesquiterpenes, α-Gurjunene, β-Cubebene, Germacrene-B)SD (linalool, hexyl hexanoate, hexyl isovalerate, hexyl-2-methyl-butanoate, sclareol, hexyl octanoate)Sh,HD (δ–Cadinen and Sclareol, Franesol, δ-Amorphene Caryophyllene oxide, Hexyl octanoate, Beta Eudesmol, α-Bisabolol, α-Muurolol, Decanoic acid, Manoyl oxide, Manool)Sh,HD (Sclareol, (+) Spathulenol, (-)-Aristolenel, β-Elemene, Hexyl n-valerate, Germacrene D, β-Eudesmol)Sh,HD (linalool, hexil isovalrate, hexil 2-methyl buterat, δ-cadinen) (piperitone)	[AM] (AM: *Streptococcus pneumoniae, Klebsiella pneumoniae, Staphylococcus aureus, Escherichia coli, Staphylococcus epidermidis*)	[140,141]
7	*Salvia mirzayanii* Rech.f. and Esfandiari	Sh	(Yellow color)Sh,HD	(2.2)Sh,HD (11.2)MW (0.50 to 9.67)SFE	HD/MW/SFE	(linalyl acetate, 1,8-cineole, linalool, 8-acetoxy linalool)HD/SFE (linalyl acetate, linalool, 1,8-cineol, 8-acetoxy, linalool, a-terpineole, E-anethole, d-cadinene)HD (linalyl acetate)SFE (spathulenol, γ-cadinene, linalool, α-terpinyl acetate, α-cadinol, β-eudesmol, cubenol, linalyl acetate)Sh,HD (α-terpinenyl acetate, 1,8-cineol, linalool)Sh (linalyl acetate, linalool, α-terpinyl acetate, 1,8–cineol, α-terpineol, δ-cadinene)HD	[AM]Sh [AB] (AM: good activity)Sh (AB: against *E.coli*, *S.aureus*, *K.pneumonia, B.subtilis, P.aeroginosa*)	[200]
8	*Salvia spinosa* L.	Sh	(Yellow color)Sh,HD	(0.2)Sh,HD (0.02)Sh, HD	HD/SD	(High amounts of monoterpene derivatives, low amounts of sesquiterpenes, phenylpropanoids, aliphatic esters)Sh,HD (thymol)Sh,HD (1,8-cineol, (z)-β-ocimene, germacrene d, 2-Butyl thiophene, trans caryophyllene, 3-Butyl thiophene)Sh,HD ((E)-β-ocimene, β-caryophyllene, isopentyl isovalerate)Sh,SD	[AB/AM]Sh,HD (AB: *Staphylococcus aureus, Basillus subtilis, Psedomonas aeruginosa*)Sh,HD	[136]
9	*Teucrium polium* L.	L/St/Sh	(Yellow color)Sh,SD	(0.8 ± 0.04)Sh, HD (0.8) (1.7) (0.2)Sh,HD (0.75)L/St,HD (1.2)Sh,HD (0.21)Sh, SD SFE	HD/SD/SFE	(Sesquiterpenes, Germacrene D, β-caryophyllene)HD/SFE (terpenoidal compounds, rich in alcohols, esters) (8-cedren-13-ol, β-caryophllene, germacrene D, sabinene)Sh,HD (α-pinene, β-pinene, p-cymene) (α-cadinol, 3β-hydroxy-α-muurolene, α-pinene, β-pinene)Sh,HD (β-pinene, limonene, α-phellandrene and γ- and δ-cadinenes. Alcohols: linalool, terpine-4-ol, cedrol, cedrenol, guaiol) (a-pinene, linalool, caryophyllene oxide, b-pinene, b-caryophyllene)L/St,HD (germacrene D, bicyclogermacrene, ß-pinene, carvacrol)Sh,SD (β-pinene, β-caryophyllene, α-pinene, caryophyllene oxide, myrcene, germacrene-D)Sh	[GP]Sh,HD [ASP] [ASP]Sh,HD [AB]HD [moderate AM]Sh,SD (AB: against *Bacillus cereus*)HD (AM: moderate effect against *Bacillus cereus, Enterococcus faecalis, Escherichia coli, Staphylococcus aureus*)Sh,SD	[137]
10	*Teucrium stocksianum* Boiss.	Sh/Fl	(Light yellow color)Sh, HD	(0.34)Sh, HD (0.4)Sh,HD	HD	(Sesquiterpenoids rich, cis-sesquisabinene hydrate rich, epi-β-bisabolol, guaiol, β-eudesmol, monoterpenoids rich)Sh,HD (a-cadinol, 6-cadinene, seychellene, P-caryophyllene, germacrene-D-4-01, germacrene D, γ-cadinene, a-muurolene, valencene)Sh,HD (camphene, α-cadinol, myrcene, carvacrol)HD (Monoterpenoids: α-pinene, β-pinene, myrcene, sabinene)Sh,HD	[AN]Sh,HD	[6]
11	*Zataria multiflora* Boiss.	L/Sh	.	(3)SD (2.8)HD (1.59 ± 0.86 to 0.99 ± 0.29)Sh, HD (1.66, 1.71, 2.8) (0.82 to 0.97)Sh,HD (3.66, 3.44)MW	HD/SD/MW/SFE	(Rich oxygenated monoterpens)Sh,HD (phenolic monoterpenes, glycosides of monoterpenes, polyhydroxy monoterpenes, benzoic acid derivatives, alkanes, β-sitosterol, betulin, fatty acids, oleanolic acid) (thymol, λ-terpinene, ρ-cymene)SD/SFE (carvacrol, thymol, p-cymene, linalool, α-terpineol) (thymol, carvacrol, para-cymene, c-terpinene, b-caryophyllene)HD (Thymol, carvacrol, ρ-cymene)Sh,HD (thymol, carvacrol, p-cymene, linalool, γ-terpinene) (γ-terpinene, α-pinene, eucaliptol, globulol)SD (oxygen-containing monoterpenes, sesquiterpene hyrocarbons, monoterpene hydrocarbons)Sh (linalol, linalyl acetate, β-caryophyllene)Sh (thymol, a phenolic compound of oxygenated monoterpens)Sh,HD (carvacrol, thymol, linalool, p-cymene)Sh,HD	[IS]Sh [AB/AO]HD [AF]SD [AF]Sh,HD [AM]Sh,SD [strong AO]Sh,HD [CE] (AB: strong activity especially against G-bacteria. *Staphylococcus aureus, Escherichia coli, Klebsiella pneumoniae, Staphylococcus epidermidis, Enterococcus faecalis, Bacillus subtilis, Salmonella typhi, Seratia marcescens, Shigella flexneri*)HD (AB: *Staphylococcus aureus*)Sh,HD (IS: against *Rhyzopertha dominica*, *Togoderma granarium*)Sh (AF: against aflatoxin by *Aspergillus flavus*)SD (AM: *Bacillus cereus*, *Salmonella Typhimurium*, *Staphylococcus aureus*)Sh,SD (AB: *Staphylococcus aureus*, *Escherichia coli*)L (G+: *Bacillus subtilis, Staphylococcus epidermidis*. G-: *Pseudomonas aeroginosa, Escherichia coli*. Pathogenic yeasts: *Candida albicans, Candida tropicalis*)Sh,HD	[141]
